# Discovery of New States of Immunomodulation for Vaccine Adjuvants via High Throughput Screening: Expanding Innate Responses to PRRs

**DOI:** 10.1021/acscentsci.2c01351

**Published:** 2023-02-23

**Authors:** Jeremiah Y. Kim, Matthew G. Rosenberger, Siquan Chen, Carman KM IP, Azadeh Bahmani, Qing Chen, Jinjing Shen, Yifeng Tang, Andrew Wang, Emma Kenna, Minjun Son, Savaş Tay, Andrew L. Ferguson, Aaron P. Esser-Kahn

**Affiliations:** Pritzker School of Molecular Engineering, University of Chicago, 5640 South Ellis Avenue, Chicago, Illinois 60637, United States

## Abstract

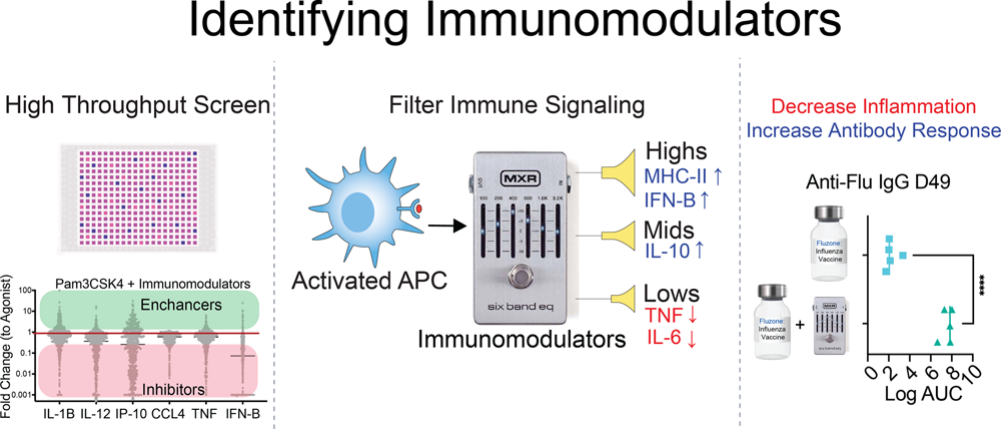

Stimulation of the innate immune system is crucial in both effective vaccinations and immunotherapies. This is often achieved through adjuvants, molecules that usually activate pattern recognition receptors (PRRs) and stimulate two innate immune signaling pathways: the nuclear factor kappa-light-chain-enhancer of activated B-cells pathway (NF-κB) and the interferon regulatory factors pathway (IRF). Here, we demonstrate the ability to alter and improve adjuvant activity via the addition of small molecule “immunomodulators”. By modulating signaling activity instead of receptor binding, these molecules allow the customization of select innate responses. We demonstrate both inhibition and enhancement of the products of the NF-κB and IRF pathways by several orders of magnitude. Some modulators apply generally across many receptors, while others focus specifically on individual receptors. Modulators boost correlates of a protective immune responses in a commercial flu vaccine model and reduced correlates of reactogenicity in a typhoid vaccine model. These modulators have a range of applications: from adjuvanticity in prophylactics to enhancement of immunotherapy.

## Abstract 2

With high-throughput screening, we identified immunomodulators that alter vaccine adjuvants. Immunomodulators reduce systemic inflammatory cytokines and increase antigen specific antibodies.

## Introduction

Vaccines are often heralded as one of the greatest triumphs of modern medicine and are a key defensive measure against infectious disease and cancer. Underneath the adaptive responses lies the stimulation of innate immune cells through pattern recognition elements. This is most often achieved via adjuvants—exogenous molecules which help stimulate innate immune pathways.^1^ In vaccines, adjuvants require a careful balance between stimulation and tolerability—excess levels of activation often result in systemic inflammation and challenges with reactogenicity.^2,3^ In immunotherapy, adjuvants face suppression from tumor microenvironments, weakening potential therapies resulting in the need to amplify interferon responses.^4^ In each of these applications, there is a need to improve adjuvant profiles by increasing or decreasing specific elements of innate signaling. Engineering individual adjuvants toward these unique circumstances, however, has proved quite challenging. Thus, we sought new approaches to modulate and tailor the immune response in the early stages of activation by altering signaling pathways.

Toward this goal, we hypothesized that manipulating the activity of two innate immune pathways, the nuclear factor kappa-light-chain-enhancer of activated B-cells pathway (NF-κB) and the interferon regulatory factors pathway (IRF), could be used to modulate innate immune stimulation.^5,6^ Signaling in these pathways begins with the binding of pattern recognition receptors (PRRs)—a common target for adjuvants and vaccine activity.^7^ When activated, these pathways develop many aspects of innate immunity: from cytokine responses to antigen presentation.^8^ However, collectively, NF-κB and IRF contain more than 100 unique proteins within their signaling network providing many potential areas beyond the PRRs for manipulation.^9^

Rather than search for novel agonists for these pathways, we explored the potential to manipulate the signaling activity of existing ligands through the addition of small molecules we term “immunomodulators”. This approach differs from prior use of small molecules in vaccine adjuvants as other high throughput screens identify small molecules that exhibit immunostimulatory activity.^10−12^ Previously, we demonstrated the possibility of modulation via a selective NF-κB inhibitor, SN50. When combined with CpG, a potent TLR9 agonist, SN50 reduced the systemic inflammatory cytokines TNF-α and IL-6 while improving antigen-specific antibody titers.^13,14^ To expand upon these results, we developed a multistep high throughput screening approach to study a library of ∼3,000 small molecule modulators in combination with a wide array of existing PRR agonists. We observed significant changes of transcription factor activity and cytokine expression in our *in vitro* screens. Modulators inhibited and enhanced both NF-κB and IRF, resulting in different activation profiles across all PRRs. Surprisingly, some modulators demonstrated activity that was broadly general across many receptors, whereas others were only effective against one or a small subset of receptors. Throughout our screening process, we developed tools to quantitatively score and select the top performing combinations of agonist and modulator with the goal of applying these toward vaccination.

Lastly, we explored the translation of our modulators to an *in vivo* setting. We identified two noteworthy classes of modulators: modulators that reduce proinflammatory cytokines and modulators that enhance antibody levels. These two classes of modulators were applied to commercial typhoid and influenza vaccines to reduce pro-inflammatory cytokines and enhance antibody levels, respectively.

## Results

### NF-κB and IRF Transcription Factor Activity Altered by Immunomodulators in a Primary Screen

To identify new adjuvants, we conducted a high throughput screen to examine differing levels of innate immune cell NF-κB and IRF activity after treatment with immunomodulators in combination with PRR agonists. We chose RAW-Dual macrophages so that both IRF and NF-κB could be measured in parallel.^15,16^ For our primary screen, we explored a targeted library of small molecules: 246 NF-κB and IRF inhibitors and 2,895 pathway specific inhibitors (SI Appendix, Table S1). Many of the included compounds were previously studied, a few even receiving FDA approval for various therapeutic applications. We hypothesized this library had an increased likelihood of modulating our desired immune signaling pathways. We tested this library’s modulation of 13 PRR agonists, with the majority being toll-like receptors (TLRs) (SI Appendix, Table S2).^17^ We included this wide range of agonists to better understand trends in modulator activity across similar or distinct PRRs and signaling pathways.

To screen this initial library for activity in modulating NF-κB and IRF activity, we seeded cells in 384 well plates using high throughput robotics. Immunomodulator compounds were added in DMSO, via pin-drop, to a final concentration of 10 μM (<0.05% DMSO vol/vol). Following 1 h incubation at 37 °C, one of 14 PRR agonists was added to approximately the EC_50_ for each agonist^a^ (SI Appendix, Table S2). Cells were incubated with agonists and modulators for 24 h and supernatant was drawn for simultaneous analysis. To ensure consistent and quality results, we optimized this workflow including: cell seeding density, incubation time, agonist concentration, liquid handling, reagent volume, and plate uniformity (SI Appendix, Figure S1).^18,19^

**Figure 1:**
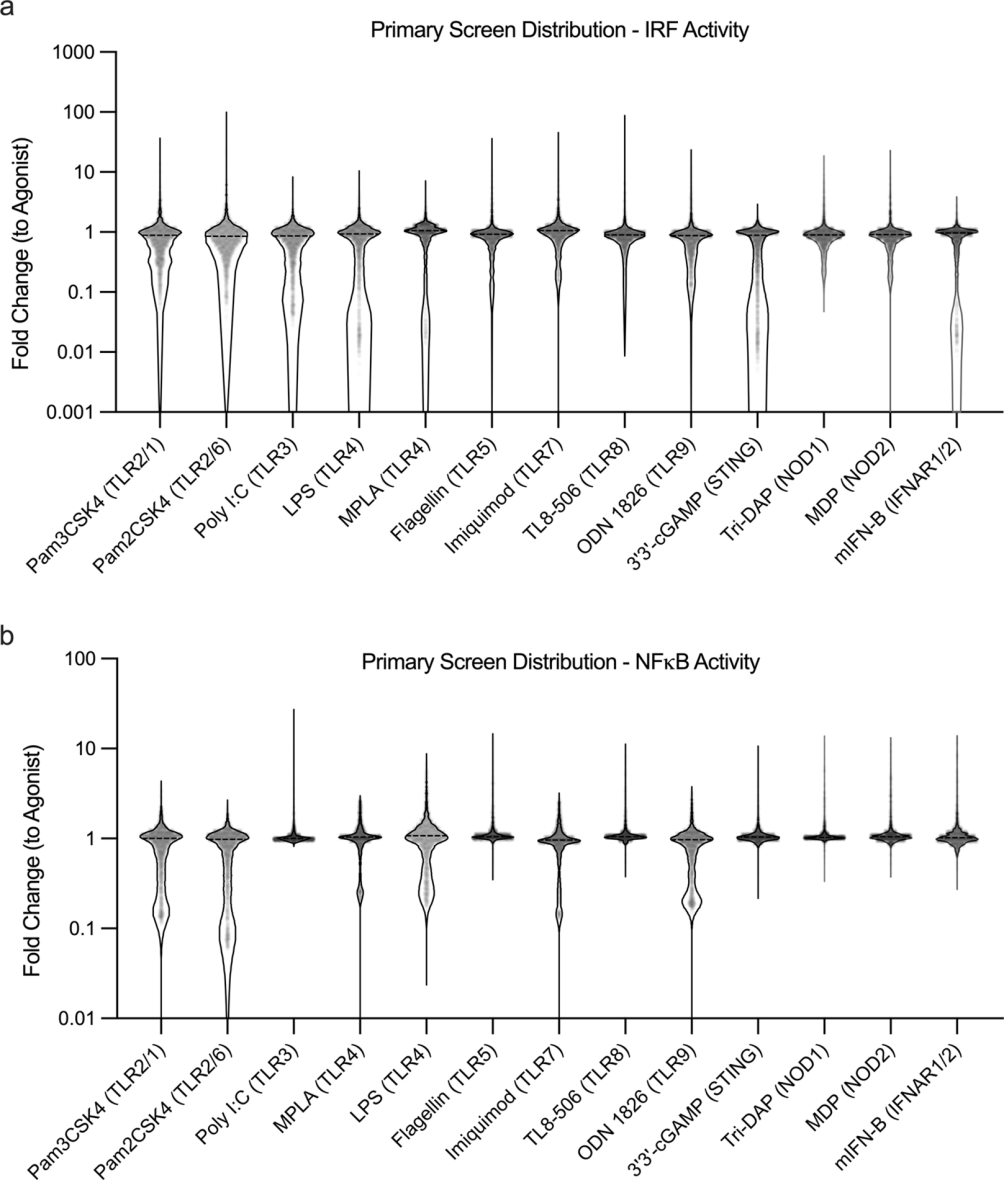
Distributions of modulated NF-κB and IRF activity from primary screen. RAW Dual cells transcription factor activity for IRF (A), and NF-κB (B) 24 h after addition of modulator + agonist. Modulator (*N* = 3147) + agonist (*N* = 13) activity reported as a fold change compared to agonist alone activity. Inhibition and enhancement over multiple orders of magnitude is observed.

Our initial screening approach presented unique challenges regarding this assay optimization and analysis. Most high throughput screens seek to either maximize or minimize a desired output.^10−12^ As such, a typical result might report enhancement as a fold-change above a baseline. In our primary screen, we anticipated finding inhibition of both immune pathways. However, we were surprised to see for the 13 agonists studied, addition of different modulators produced either enhancement or inhibition—sometimes ranging over 100-fold in both directions compared to agonist alone controls (Figure 1). This modulation persists even when using potent agonists with high levels of activity. For example, modulation of 3′3′-cGAMP, a STING agonist, showed a 5-fold increase in IRF activity—a result which surprised us as very few molecular entities have achieved higher activation of STING than 3′3′-cGAMP.^20^ The balance of measuring large degrees of both inhibition and enhancement tested the limit of the assay’s dynamic range—an issue that persisted throughout our various screening efforts.

### Classifying Immunomodulators on PRR Agonist Trends

**Figure 2:**
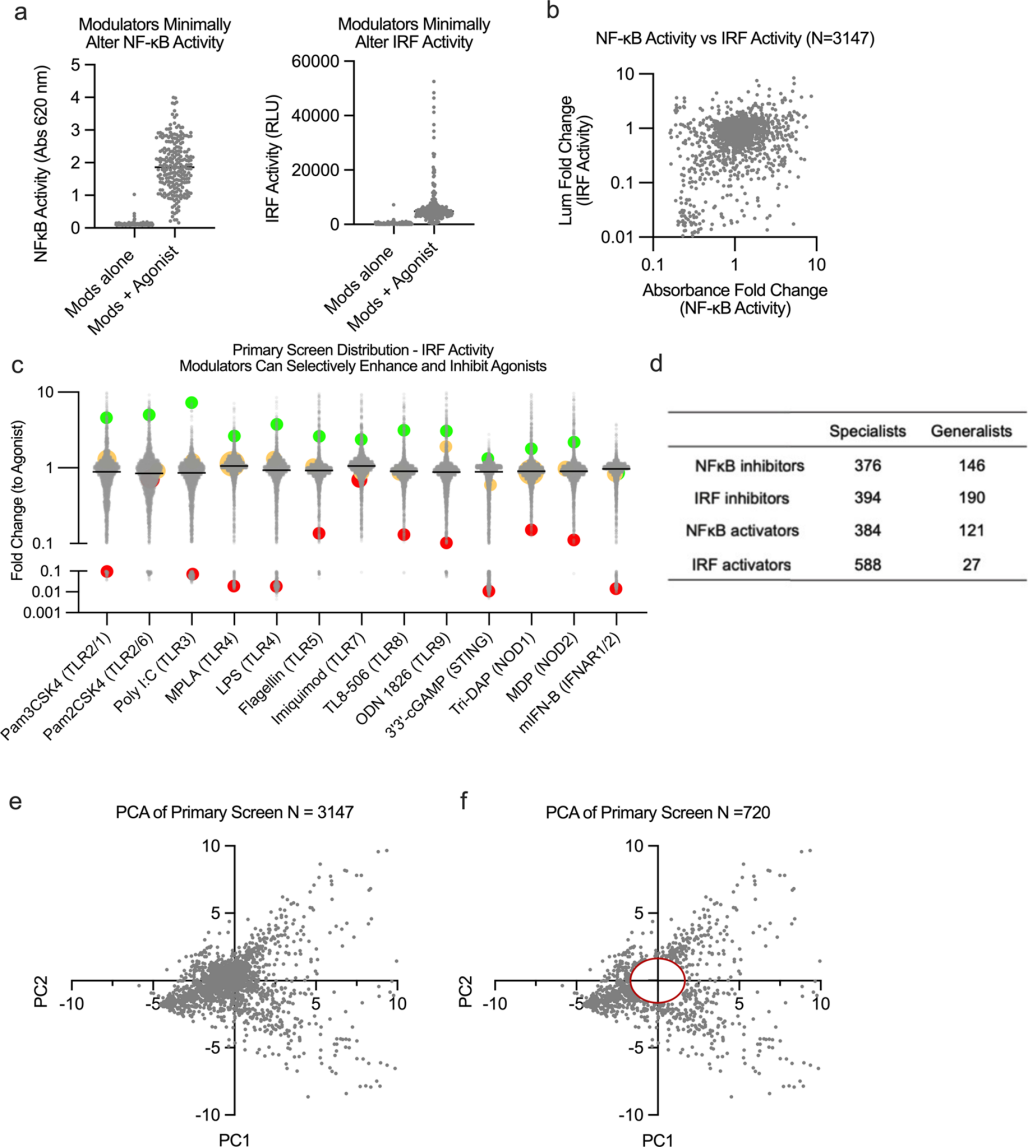
Primary screen trends and down selection process (A) Modulators (*N* = 3147) alone show little inherent activity in either NF-κB or IRF transcription factors. (B) Modulation of transcription factor for NF-κB with LPS shown to act independently of IRF transcription factor modulation (*R*^2^ = 0.0409). (C) Modulators demonstrate different trends across agonists: general enhancers (green), general inhibitors (red), or specialist activity (yellow). (D) Table summary of specialist modulators (active with only one agonist) and generalist modulators (active with 12–13 agonists). (E) Principle component analysis with IRF and NF-κB transcription factor data from all modulators (*N* = 3147) and high *z*-factor agonists (*N* = 8). (F) Compounds with minimal variability were removed by creating a circle centered on the origin with radius 1.75.

Having successfully completed our primary screen, we began to study additional aspects of modulator activity with the goal of identifying optimal modulators for further study. First, we ensured that modulators alone do not exhibit inherent stimulation of either NF-κB or IRF, but rather that only combinations of modulator and agonist lead to variation in transcription factor activity (Figure 2A). Compounds that significantly activated either pathway on their own were removed from further study (<1% of the library). Upon comparing NF-κB and IRF transcription activity, we observed little correlation between the two (*R*^2^ = 0.0409), indicating these pathways can be studied independently with our assay (Figure 2B).

A key observation from this larger data set was that modulators act either specifically or generally. For example, *modulator X* enhances IRF for TLR4, while other PRRs’ activities remain unaffected. Conversely, *modulator Y* enhances IRF for all receptors. To identify each type of modulation, we classify immunomodulators that are specific to a few receptors as “specialists” and modulators that affect nearly all receptors as “generalists” (Figure 2C, D). Further, some modulators enhanced one PRR for a particular pathway and yet inhibited another PRR for the same pathway. We see wide distributions across each receptor/pathway, with some agonists showing greater statistical significance due to a larger dynamic range. Monitoring distributions across similar PRR targets revealed a correlation in their activity. For instance, modulation of MPLA and LPS, both TLR4 agonists, showed similar trends across NF-κB and IRF activity. Modulation of Pam2CSK4 (TLR1/2), Pam3CSK4 (TLR2/6), and other NF-κB dominant agonists also have degrees of correlation (SI Appendix, Figure S2).

### Removal of Inactive and Undesirable Modulator/Agonist Combinations

We sought to identify high modulatory compounds while removing inactive or toxic modulator/agonist combinations. We first designed a high throughput method to filter toxic modulators by measuring viability. As a proxy of viability, we used live cell imaging combined with digital analysis to create confluency masks (SI Appendix, Figure S3).^21,22^ After applying our viability masks, we identified compounds with the highest likelihood of altering IRF and NF-κB responses. To focus on high value PRR/modulator pairings, we also removed PRR agonists based on a lower Z-factor cutoff score (SI Appendix, Table S3).

In this down selection stage, we did not yet prioritize enhancement or inhibition, but sought only to remove compounds that had minimal effects on PRR agonist activity. To discern the relative level of activity, we employed principal component analysis on the data set to quantitatively compare levels of variance between the compounds.^23^ This data set included both NF-κB and IRF distributions from eight agonists for a total of 16 variables. PC1 and PC2 accounted for 49% of the variation within our data set. We sought to move forward only compounds that had major changes to immune response, so we created a circle with a radius of ∼1.75 PCA units centered on the origin. We rationalized this because, when using PCA, data points centered around the origin contain the least amount of variability. We retained compounds outside this radius—reducing the number of immunomodulators from 3,147 to 720 (Figure 2E, F). This numerical cutoff was chosen to maximize the number of dynamic modulators compared in higher cost multiplexed cytokine responses which we sought to correlate with tolerability and efficacy. These 720 compounds composed our secondary library for further screening analysis, preserving the high degree of pathway modulation (SI Appendix, Figure S4).

### Cytokine Expression Changed by Immunomodulators in a Secondary Screen

Our next goal was to determine how the modulators would alter the cytokine response of innate immune cells to identify compounds for use in *in vivo* experiments. Cytokines and chemokines are secreted signaling proteins induced by adjuvants to regulate adaptive immunity.^24^ However, excessive production of certain cytokines by adjuvants results in tissue damage. This response is strongly correlated with vaccine tolerability.^25,26^ To achieve the wide dynamic range necessary for modulators, *in situ* measurement, and multiplexed measurement, we employed the AlphaLISA assay.^27−29^ As we narrowed our compounds, we sought to ensure their compatibility with human immune responses. Conveniently, human AlphaLISAs provided more multiplexed cytokines. As a result, we performed the screen with THP-1 monocytes. We measured the levels of six cytokines/chemokines involved in inflammation, tolerability and adaptive responses—IL-12p40, IP-10, IL-1, CCL4, TNF-α, and IFN-β—accounting for a wide dynamic range and assay metrics (SI Appendix, Table S4).^30^^b^ TNF-α and IL-1β are endogenous pyrogens, and there are multiple reports correlating them with induction of fever for vaccine tolerability.^31^ TNF-α provided a baseline measure of generalized inflammation. Additionally, IL-1β is a measure of inflammation that is partially outside direct NF-κB regulation, unlike TNF-α, enabling us to differentiate compounds based on their pathways of inflammation.^32^ We chose to study IFN-β due it strong correlation with IRF pathway and its role as an antiviral type I interferon.^33^ IL-12/23p40 activates NK cells, induces production of IFN-γ, and polarizes toward a Th1 and Th17 response.^34^ IP-10 (CXCL10) is a chemoattractant for T cells and DC cells and correlated in adjuvants studies as an early signal of induction of strong responses.^35^ Finally, CCL4 is a chemoattractant for monocytes and NK cells.^36^ IP-10 and CCL4 were recently shown to correlate with tolerability issues.^37^

**Figure 3:**
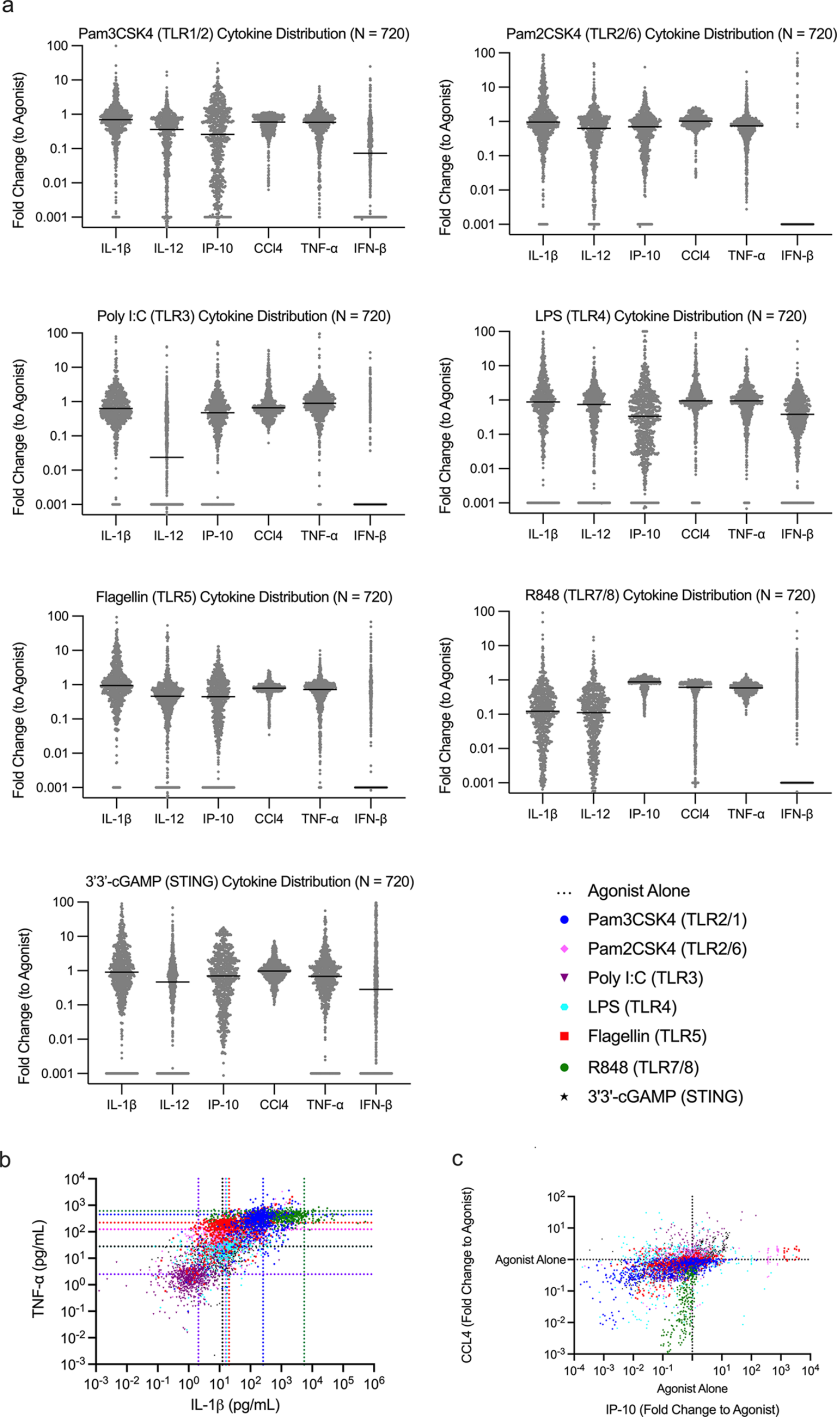
Distributions of modulated cytokine expression from secondary screen. (A) THP-1 cytokine distributions for TNF-α, IL-1β, IFN-β, IL-12/23 (p40), IP-10 (CXCL10), and CCL4 24 h after addition of modulator + agonist. Modulator (*N* = 720) + agonist (*N* = 7) activity reported as a fold change compared to agonist alone activity. Inhibition and enhancement over multiple orders of magnitude is observed. Nondetectable measurements of cytokines were given a value of 0.001 fold change. (B) Concentrations of TNF-α and IL-1β (pg/mL) of each agonist + modulator combination. Dotted lines represent agonist alone secretion. Modulation allows for enhancement and inhibition independent of cytokines. (C) Modulation of CCL4 and IP-10 of each agonist + modulator combination, represented as a fold change compared to agonist alone activity.

This cytokine assay, the secondary screen, had an identical workflow as the primary screen until supernatant analysis. Cell supernatants were collected, and cytokines were measured in three, duplexed measurements (SI Appendix, Figure S5A). We optimized standard curve ranges, crosstalk correction factors, incubation times, and other parameters of the secondary screen to account for any differences between experiments (SI Appendix, Figure S5B–D). We stimulated cells with a subset of seven agonists from the primary screen (SI Appendix, Table S4). Modulators enhanced or inhibited cytokine production among all six cytokines by several orders of magnitude (Figure 3A). Similar to the primary screen, we observed that modulators could alter signals independent of one another (Figure 3B, C). The distributions within cytokines varied based on the dynamic range and *Z*-factor obtained for each cytokine and agonist studied (SI Appendix, Table S5). Since each agonist produced differing levels of cytokine, sometimes near the limits of the standard curve, amending this assay for high-throughput analysis had limitations. Since the assays were multiplexed, dilution of individual wells or selection of other AlphaPlex excitation/emission profiles would increase cost and time significantly. This resulted in some compression of cytokine responses with lower or higher levels: notably, IFN-β, which had a relatively low signal, and IP-10/CCL4, which had high signal and concentrations. Since we measure fold-change and reduce dimensionality in analysis, this approach is adequate to compare compounds within our data set for downselection. However, on account of these limitations, we caution the reader not to interpret any individual compound’s cytokine response as equivalent to a typical ELISA assay with exacting parameters.

Similar to our primary screen, we observed that modulators alone do not natively affect or induce cytokine release, but rather the combination of agonist and modulator elicits a large increase or decrease in cytokine and chemokine production (SI Appendix, Figure S6). Changes in cytokine activity did not always correlate with a corresponding level of change in the transcription factor activity for the same modulator. We did observe that, for the most active compounds, transcription factor activity correlated with an increase or decrease in cytokine response. For example, the strongest inhibitors of NF-κB also resulted in the lowest TNF-α levels (SI Appendix, Figure S7A). While the modulators can alter responses in unique patterns, they appear to do so with a generalized conservation of signalized pathways. When comparing agonists with similar profiles such as Pam2CS4K and Pam3CSK4, similar trends of enhancement and inhibition for each cytokine are observed (SI Appendix, Figure S7B).

To validate the effectiveness of our downselection from the primary screen, we compared cytokine modulation between the selected compounds and an equivalent, random portion of the original primary screen library. We selected LPS as the agonist on account of its wide dynamic range. The secondary, downselected library contained compounds with far greater TNF-α range of activity compared to the random, equal in number primary screen compounds evaluated using an F test comparing Kurtosis of log-transformed TNF-α values (SI Appendix, Figure S8) supporting using NF-κB and IRF activity as a valuable downselection tool.

### Defining Top Candidates via a Flexible, Quantitative Scoring System

With an increasing number of variables to consider when searching for desirable agonist/modulator combinations, we sought to develop a general framework to assist in the final down selection of modulators for testing in various *in vivo* applications. Since our previous work focused on improving adjuvants for prophylactic vaccines, we developed our first scoring system to identify candidates for this use—creating a “vaccine score” as a quantitative metric.

**Figure 4:**
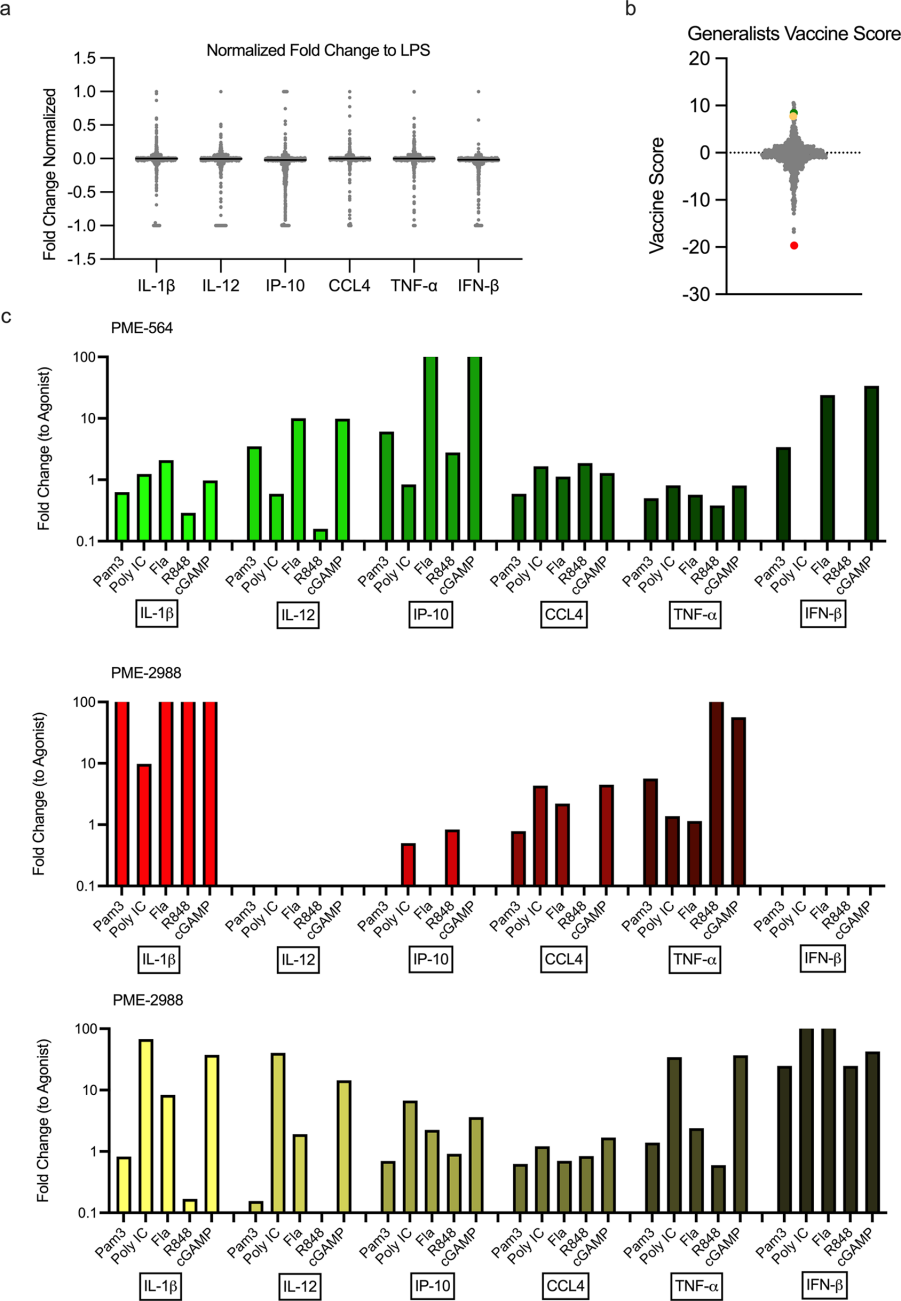
Demonstration of modulator analysis through a “vaccine score”. (A) Representative normalized cytokine distributions for one agonist, LPS (*N* = 720). (B) Fold changes for cytokines were multiplied by weight factors and summed to obtain agonist specific “vaccine scores”. All agonist scores were combined to create the generalist score (*N* = 720). (C) Cytokine production of a top vaccine score candidate, PME-564 (green), a negative score candidate, PME-2988 (red), and a candidate for additional applications, PME-2539 (yellow). Modulator + agonist activity reported as a fold change compared to agonist alone activity.

Modulator performance was quantified such that the “vaccine score” would preserve cytokine changes by normalizing each cytokine’s and agonist’s dynamic range.^38^ To account for differences in the dynamic range of all six cytokines, we normalized the data to ensure no single cytokines distribution would bias the results. Thus, cytokine responses were transformed to fit a range from −1 to 1 (Figure 4A). Unlike our previous screen, we considered increases and decreases in cytokine responses separately when selecting molecules for vaccination studies. In a potential vaccine, a promising candidate would need to produce minimal systemic pro-inflammatory cytokines while increasing IFN-β and chemokine production.^25,26^ Additionally, the score might prioritize the importance of one cytokine’s modulation over another. To account for each of these issues, we assigned weighting variables of varying magnitudes to each cytokine depending on the desired modulatory effect (SI Appendix, Figure S9A).^39^ Because modulators acted on individual receptors with distinct responses, the first result from the vaccine scoring system is a “specialist” score for a specific agonist + modulator combination (SI Appendix, Figure S9B). To then identify modulators which improved responses across multiple PRRs, the individual scores were summed to provide a “generalist” score across all agonists (Figure 4B).

The result of the vaccine scoring methodology created a spread among compounds ranging from 10 at the highest to nearly −20 at the lowest. Within the highest rated compounds for generalist modulators, the scoring system resulted in approximately 20 with scores between 6 and 10 from the total pool of 720 potential compounds. As a representation, we included one example compound to demonstrate the patterns observed for the individual modulators—PME-564 (Figure 4C). PME-564, a tyrosine kinase inhibitor, was one of the highest scoring compounds on our ranking system. Its high score can be attributed to a strong enhancement of IP-10 across most agonists and enhancement of IFN-β and IL-12 for several agonists. PME-564 also decreased TNF-α expression across all agonists with notably high suppression for Pam3CSK4 and Resiquimod (R848) (Figure 4C). In selecting for a “generalist”, we note that modulator activity is not exactly equal across all agonists. For example, comparing PME-564’s enhancement of IP-10 and IFN-β for Pam3CSK4, Pam2CSK4 vs cGAMP, there are nearly 2 orders of magnitude in difference. This suggests that depending on the application, a generalist might still have limitations vs specialist modulators for enhancing specific pathways. However, for suppression of inflammatory signals and thereby its ability to promote tolerability, this category of modulators was broadly general. Suppressing inflammatory cytokines, even partially, for nearly every PRR suggests these molecules might be used toward improved tolerability and broad use in improved vaccination or other immunotherapies.

In contrast to PME-564, we highlight PME-2988 (Figure 4C), which is a compound in our data set with a strong negative vaccine score. This compound eliminated IL-12, IFN-β, and IP-10 secretion across most receptors studied while simultaneously enhancing IL-1β and TNF-α secretion by nearly 100-fold. PME-2988 will not be useful as a vaccine adjuvant, but its ability to radically alter secreted cytokines highlights the wide-ranging potential of modulators. This score is tailored to identify prophylactic vaccine adjuvants, but compounds within this data set may be applicable for exploration in alternative applications. For instance, PME-2539 (Figure 4C), may warrant additional study in a cancer immunotherapy as it upregulates beneficial antitumor cytokines and chemokines.^40,41^ This compound can enhance TNF-α up to 36-fold using cGAMP and is shown to enhance IFN-β secretion across all agonists studied. While further enrichment of cancer adjuvants is outside the scope of our current work, we seek to investigate these applications more in the future.

We used the general vaccine score to identify a small subset of lead modulators of interest for preliminary *in vivo* studies. These compounds were also validated in murine bone marrow-derived dendritic cells (BMDCs). These immunomodulators altered cytokine production and cell surface markers in combination with multiple agonists (SI Appendix, Figure S10). The foundation and simple mathematical nature of our scoring system allows us to apply this methodology to different areas of interest, albeit limited by the scope of the cytokine panel studied. Additionally, further screening efforts such as cell surface marker expression could be incorporated into this scoring system in the future. While we created a vaccine scoring system, a similar methodology could be tailored toward other applications, whether for inflammation therapeutics or cancer immunotherapy, control of immune pathways has potential in many immune-therapeutic spaces.

### Immunomodulator NF-κB Activation Dynamics

As NF-κB activation regulates many of the cytokines used in the panel, we then looked at whether NF-κB activation dynamics were altered by treatment with our top compounds. Altered activation dynamics might reveal how top compounds reshape the signaling pathways of NF-κB activation. We differentiated bone-marrow-derived macrophages (BMMΦ) from endogenously tagged RelA-YFP mice, loaded them into a custom microfluidic device,^42^ and tracked RelA nuclear translocation in single BMMΦ over treatment with six modulators chosen with high vaccine scores ± R848. We observed that modulators alone resulted in negligible activation of RelA (SI Appendix, Figure S11A), while treatment with R848 induced rapid nuclear translocation of RelA (SI Appendix, Figure S11B). We then looked at specific features of NF-κB activation over time, namely, peak amplitude, area-under-the-curve (AUC), response duration, and late AUC (SI Appendix, Figure S11C), which correspond to distinct epigenetic and transcriptional states in activated cells.^43^ Late AUC was significantly increased following treatment with PME-564 and PME-2809, suggesting that these compounds may alter transcriptional feedback or receptor inactivation (SI Appendix, Figure S11G). In general, however, we saw that other features were similar between untreated and treated conditions (SI Appendix, Figure S11D–F). Thus, although these compounds modulated NF-κB regulated cytokine production, they appeared to act independently of NF-κB activation. This observation raises the possibility that these modulators act on synergistic pathways or at the level of transcription.

### Identified Candidates Improve Vaccination Responses in Mice

**Figure 5:**
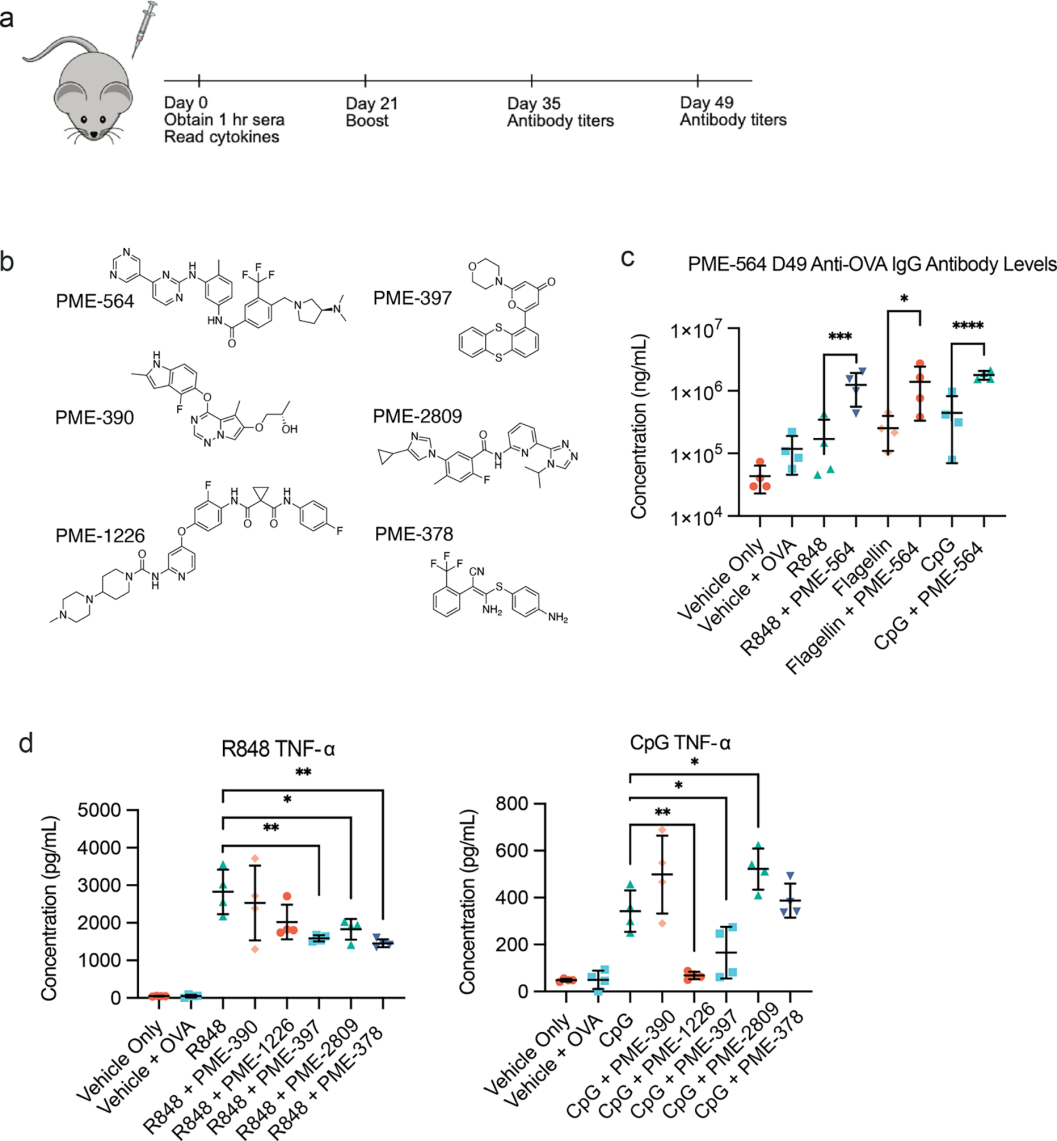
*In vivo* immunomodulation improves adjuvants in a model ovalbumin vaccination. (A) Schematic of *in vivo* study timeline. (B) Additional modulators studied *in vivo* and their structures. (C) Agonist + PME-564 serum anti-OVA IgG antibody levels, day 49, *n* = 4. Statistical analyses between agonist and agonist + PME-564 were performed by an unpaired *t* test. (D) Systemic TNF-α levels 1 h after vaccination with agonist, agonist + modulator, and vehicle (*N* = 4) for R848 (TLR7/8) and CpG (TLR9). Statistical analyses between agonist + modulator groups and agonist alone were performed by a one-way ANOVA test *P < 0.05, **P < 0.01, ***P < 0.001.

We used our vaccine score to select modulators to test in a murine *in vivo* model of vaccination. We repeated the traditional prime-boost vaccination schedule as in our previous preliminary studies using ovalbumin as a model antigen.^13,14^ To test the generalist nature of the modulators, these subunit vaccinations were adjuvanted with a subset of the PRR agonists from our primary screen: R848 (TLR7/8), flagellin (TLR5), and CpG 1826 (TLR9). This subset was selected both for the previous use in vaccines and for a broader cross section of potential use in both subunit (R848, CpG) and as an approximation of whole bacterial (flagellin, CpG) vaccine products.^44−46^ We chose a modulator dosage of 1.5 μmol, guided by our previous experience with small molecules and compound solubility limitations.^14^ To improve formulation of these hydrophobic compounds, we used a 1:1 mixture of DMSO:Addavax as a vehicle.^c^ We monitored inflammatory cytokine levels 1 h following initial injections and measured antigen specific antibody levels at the indicated time points postboost (Figure 5A).

Our goal, as previously, was to find compounds that would (a) increase tolerability of the vaccine formulation which we approximate using a simple metric of systemic cytokines 1 h after injection and (b) improve antibody responses to the vaccination.^d^ We began by experimenting with PME-564, the highest performing compound from our generalist scoring system. The addition of this modulator resulted in an increased humoral response for all three agonists tested (Figure 5B). Addition of PME-564 improved the antibody response for these adjuvants ranging from 2- to 6-fold over their agonist/antigen controls. This increase was remarkably consistent across all the agonists and persisted across both time points (SI Appendix, Figure S12A). We did not, however, observe significant systemic cytokine modulation with this modulator (SI Appendix, Figure S12B).

Emboldened by these positive results, we expanded our search to 5 more high scoring generalists following the same vaccination schedule as before (Figure 5C). We observed that for the 5 modulators, PME-1226 and PME-397 strongly decreased TNF-α for CpG 1 h post injection (Figure 5D). This decrease in systemic inflammatory cytokines is strongly correlated with improvement in clinical scoring, temperature drop, and weight-loss providing strong indications that these compounds could be used to improve the tolerability of CpG in further applications. Interestingly, there were similarities between the compounds that reduced inflammatory cytokines for CpG and for R848. However, as with our previous experiments, modulators were unable to completely remove the inflammatory nature of R848.^13^ We hypothesize R848 diffuses rapidly away from the injection site due to its small molecular weight, inducing larger systemic effects. Yet PME-378 still reduced the inflammation to half the original formulation. No compounds showed statistically significant reduction in cytokines for flagellin, though PME-378 showed a decreasing trend (SI Appendix, Figure S13A). Reduction of inflammatory cytokines did not result in an increase in antibody levels in most cases, but instead maintained a response similar to the agonist control (SI Appendix, Figure S13B, C).

**Figure 6:**
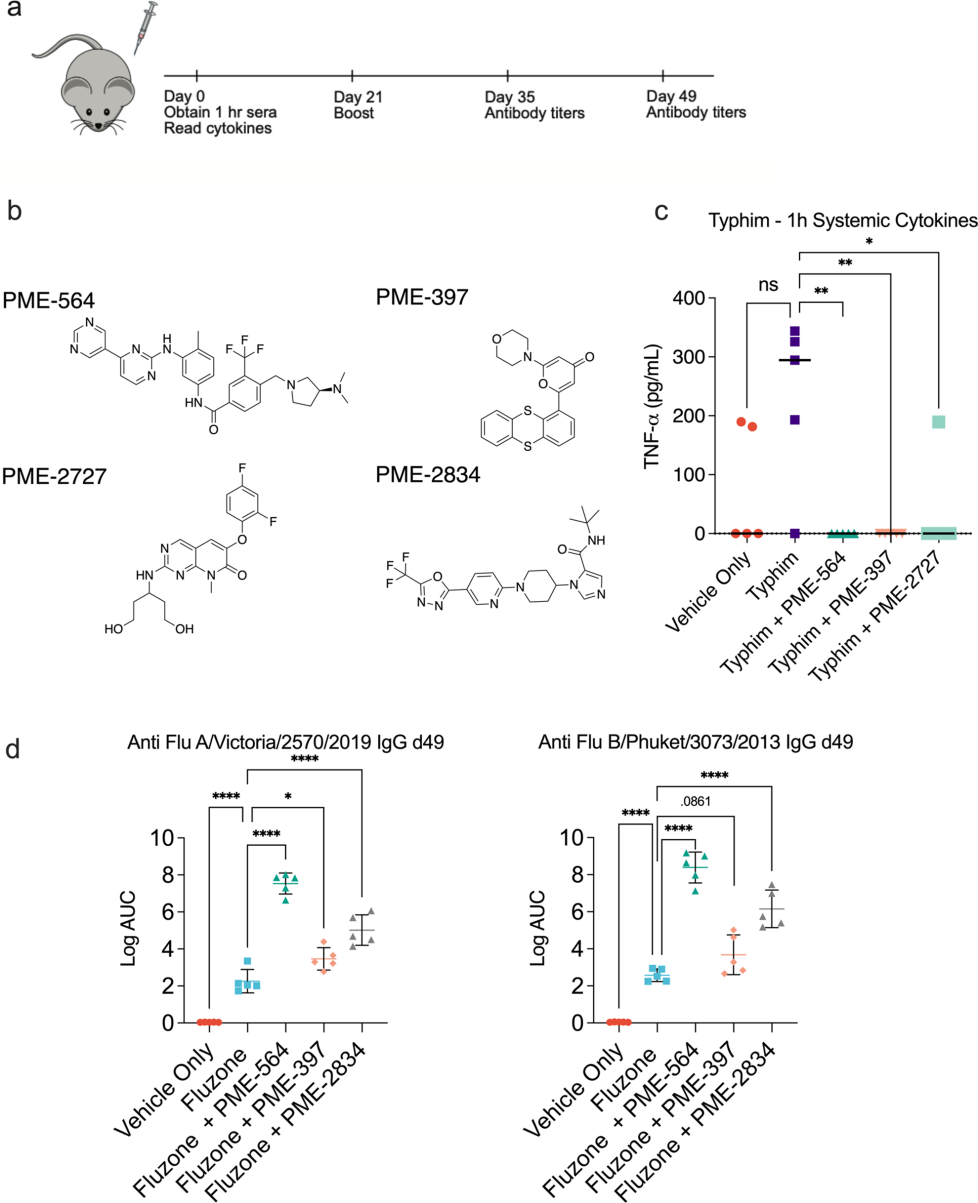
*In vivo* immunomodulation improves antibody titers in an influenza model and reduces systemic cytokines in a Typhim Vi vaccination. (A) Schematic of *in vivo* study timeline. (B) Structure of additional modulators. (C) Typhim Vi + modulator systemic TNF-α levels 1 h after vaccination with vaccine, vaccine + modulator, and vehicle (*N* = 5). Statistical analyses between agonist + modulator groups and agonist alone were performed by a one-way ANOVA test *P < 0.05, **P < 0.01. (D) Fluzone 2021–2022 + modulator antigen specific serum IgG antibody levels, day 49, *n* = 5. Statistical analyses between Fluzone and Fluzone + PME-564 were performed by a one way ANOVA test. *P < 0.05, **P < 0.01, ***P < 0.001, ****P <.0001.

Next, we investigated the addition of top modulators to two commercial vaccines: the inactivated quadrivalent influenza vaccine, Fluzone (2021–2022), and the carbohydrate based typhoid vaccine, Typhim-Vi. We administered intramuscular (i.m.) injections in C57/B6 mice that correspond to approximately 1/10th a human dose in a 21 day prime/boost injection schedule.^13,47^ We again measured systemic pro-inflammatory cytokines 1 h after the primary injection and measured antigen-specific antibody levels 4 weeks post boost (Figure 6A, B). With Typhim-Vi, we identified three modulators that significantly reduced the inflammatory cytokine TNF-α, though the response to the vaccine only was smaller than our previous vaccination studies (Figure 6C). With influenza, we measured IgG titers against hemagglutinin (HA) from two strains included in the quadrivalent vaccine: A/Victoria/2570/2019 and B/Phuket/3073/2013. We observed three modulators that statistically improved antibody titers when compared to Fluzone alone with the most notable modulator, PME-564, resulting in a ∼4-fold increase in the log AUC (Figure 6D). We then quantified cell types critical for antibody production via flow cytometry, focusing on plasmablasts, germinal center B cells, and T follicular helper cells. We did not find any significant differences in these populations between Fluzone alone and Fluzone with modulator (SI Appendix, Figure S14A–G). These results are consistent with our previous work with immunomodulators as adding SN50 to Fluzone also did not affect cell frequencies. PME-564 elicited a stronger antigen-specific antibody response than SN50 (SI Appendix, Figure S14H). Despite having no inherent innate activity on their own, these modulators modified the response of multiple commercial systems without increasing inflammatory responses. This stands in contrast with traditional adjuvants. We plan to explore modulation of vaccine systems at great depth in the future.

After multiple rounds of investigation, we have identified two classes of lead modulators: inflammatory cytokine reducing modulators as well as IgG antibody enhancing modulators. This is contrast to our previous work where these two measured values—systemic cytokine modulation and antibody responses—were directly coupled. PME-564, the modulator that enhanced antigen-specific IgG levels, only partially downregulated systemic inflammatory cytokines depending on the adjuvant. Conversely, modulators that alter systemic cytokine levels were minimally impactful on the humoral response.

While these results are promising, we hypothesize that solubility and biodistribution of our modulators may limit their effectiveness in these formulations, and optimizing formulations for our lead candidates is an active area of investigation. Initial attempts at formulating antigen, agonist, and modulators in liposomal delivery systems diminished cytokine and antibody responses across both controls and treatments (SI Appendix, Figure S15). Additional *in vivo* screening of candidate modulators is needed to further prove the efficacy of our quantitative scoring systems. While there is much more that can be learned both about the immunological mechanism and the application to specific therapies of these modulators, our efforts here focused on the screening and downselection of novel compounds with an extensive PRR library. With the identification of new compounds with new properties, we plan to examine the biological mechanism and potential for application in future studies.

## Discussion

Adjuvants and immune potentiators can enhance the immunogenicity of vaccines and immune therapies and are critical for effective clinical translation. Yet, there are currently few ways to control reactogenicity and tolerability or to enhance and suppress inflammatory and stimulatory responses. In this work, we present a high-throughput screen which identifies a new family of compounds, we term immunomodulators, that work in combination with traditional adjuvants as signal amplifiers/suppressors. We created a set of selection criteria for identifying molecules which themselves elicit minimal response, but when combined with a PRR stimulating adjuvant, result in changes to the immune response of more than an order of magnitude. This differs from traditional adjuvant discovery, in which small molecules are screened for their inherent ability to agonize receptors. With our approach, we expand innate responses to PRRs, discovering new phenotypes with unique signaling profiles. We screened molecules via a series of *in vitro* assays; first examining the modulators’ ability to alter NF-κB and IRF expression signatures, then examining their ability to alter cytokine response. We developed a ranking system to identify potential lead compounds for use *in vivo*. Through this series of down selecting primary and secondary screens, we identified a landscape of immunomodulators that can both enhance and suppress cytokine and chemokine production. Using the ranking system, we identified key modulators that lowered systemic inflammatory cytokines and increased antibody response against OVA in a vaccination experiment.

Our results demonstrated that modulators can be identified which operate with generality—improving the antigen specific antibody response more than 5-fold when used with multiple adjuvants. Conversely, modulators can also be selected which operate with specificity—matching with one adjuvant most successfully to lower systemic cytokines. The conclusion for this initial screen is that a ranking and assessment screen was sufficient to help identify modulators with a success rate *in vivo* of approximately 10–20%. While our *in vivo* results are preliminary for and much remains to be tested prior to use in clinical vaccines, modulators hold promise to enhance immune responses and mitigate side effects. At the same time, we want to highlight that this screening and selection process can be applied to modulatory outcomes for many immune-therapeutic applications beyond just vaccination.

Our study identifies a class of immunomodulators that can affect both innate and adaptive immunity. Many of these compounds have been previously used in alternative applications. For instance, our top compound, PME-564, has been used clinically in treatment against myelogenous leukemia. It inhibits the activity of multiple kinases, but it has previously demonstrated interaction with Lyn kinase. Lyn is a Src-family kinase whose roles in innate signaling extend throughout the innate system.^48^ TLR pathways are partially regulated by Lyn as the kinase is membrane bound and associated with the TLR/MyD88 complex. In pDCs, Lyn promotes the trafficking of CpG from the extracellular space to internal endosomes—altering the production of proinflammatory cytokines and Type I IFNs.^49^ Adding a Lyn inhibitor in combination with a TLR agonist may regulate proinflammatory cytokines and chemokines that we measured in our high throughput screens. While these previous studies support our findings, no Src or Lyn inhibitors had ever been combined with adjuvants in the context of vaccines. Another top modulator, PME-2834, is a pan-WNK kinase inhibitor and was originally discovered through work on hypertension.^50^ Recently, WNK kinases have been implicated in a diverse array of signaling pathways, including NF-κB.^51^ We posit this finding highlights the value of an empirical screening approach to the discovery of new modulators of innate signaling Thus, we believe existing libraries can be used to explore additional applications for drugs with known indications.

In future work, we plan to explore both the mechanistic details of how chemically distinct modulators can achieve general patterns of altered immune response. In parallel, we plan to explore how these identified compounds can be used to improve current vaccines, vaccine candidates, or immune therapies. A host of potential approaches could be enabled by employing modulators alongside current technologies including: expanding therapeutic window by increasing tolerability, increasing the overall efficacy via improved humoral responses, altering specific cytokine/chemokine responses to adjust temporal responses of vaccination.

## Supporting Information Available

Primary and secondary screening data (ZIP)
Detailed methods, key resources tables, supplementary text, high throughput workflow and optimization, BMDC immunomodulation experiments, NF-κB activation dynamics, flow cytometry analysis, extended data sets, and preliminary liposome experiment data, and compound library sources, agonist concentrations, and high throughput optimization (PDF)
Transparent Peer Review report available (PDF)

## Footnotes

a Concentrations were occasionally adjusted away from the EC_50_ if it was determined that the modulatory range was larger than expected.

b Several cytokines with excellent *in vivo* correlates did not have strong enough signals including IL-27 and IL-6.

c While Addavax, a squalene-based oil-in-water emulsion has inherent adjuvating properties, we determined its use appropriate because (a) our experiment was testing the modulation of preexisting adjuvants which would be need to be formulated in their administration and (b) we could control for inherent alterations to their response via separate adjuvant only (no modulator) controls.

d Our focus in this work was to validate our screening approach, not to explore the mechanism of the modulators studied. While this is outside the scope of this project, we did address possible mechanisms in our original modulation work.^13,14^
