# Supplementary material for: Discovery of New States of Immunomodulation for Vaccine Adjuvants via High Throughput Screening: Expanding Innate Responses to PRRs
Source: ACS Cent Sci. 2023 Feb 23;9(3):427–39. doi: 10.1021/acscentsci.2c01351 (PMC10037445; doi:10.1021/acscentsci.2c01351)
Supplement: Supplementary file 2 — oc2c01351_si_002.pdf [file oc2c01351_si_002.pdf]

Supplementary Information for

Discovery of New States of Immunomodulation for Vaccine Adjuvants via High Throughput Screening: Expanding Innate Responses to PRRs

Jeremiah Y. Kim°, Matthew G. Rosenberger°, Siquan Chen, Carman KM IP, Azadeh Bahmani, Qing Chen, Jinjing Shen, Yifeng Tang, Andrew Wang, Emma Kenna, Minjun Son, Savaş Tay, Andrew L. Ferguson, Aaron P. Esser-Kahn*
Email: aesserkahn@uchicago.edu

Pritzker School of Molecular Engineering, University of Chicago, 5640 South Ellis Avenue, Chicago, Illinois 60637, USA.
° = these authors contributed equally to this work
*Correspondence to: aesserkahn@uchicago.edu

This PDF file includes:

Materials and Methods
Key Resources Table
Supplementary text
Figures S1 to S15
Tables S1 to S5

Materials and Methods

Lead Contact
Further information and requests for resources and reagents should be directed to and will be fulfilled by the lead contact, Aaron Esser-Kahn (aesserkahn@uchicago.edu).

Materials Availability
This study did not generate new unique reagents.

Data and Code Availability
Any additional information required to reanalyze the data reported in this paper is available from the lead contact upon request.

Safety Statement
No unexpected or unusually high safety hazards were encountered.

NF-κB and IRF Transcription Factor Screening
RAW-Dual cells (InvivoGen) were plated at 50,000 cells per well in 45μL of DMEM with 5% HI FBS from col 2-23 in clear flat bottom 384 well plates (Greiner Bio-One). Cells attached at room temperature for 1 h. 50 nL of 10 mM modulator libraries were added by Janus G3 via pintool to experimental wells (cols 3-22) for a final concentration of 10 μM. Following 1 hour incubation, 5 μL of PRR agonist was added via a MultiDrop Combi liquid handler (col 3-23). Cells were incubated at 37 ˚C and 5% CO_2 overnight. 20 hours later, 12.5 μL of QuantiLuc Plus was plated in an opaque, white 384 well plate. 5 μL of RAW-DUAL cell supernatant was then added via Multi-Drop Combi Liquid handler before measuring luminescent values on a BioTek Synergy NEO2 plate reader as soon as plate was completed. QuanitLuc Plus contains a stabilizer which reduces signal decay, allowing for comparable values throughout the read. In parallel, 15 μL of 5X concentrated QuantiBlue was added directly to the remaining cell supernatant in the RAW-Dual cell plate. Absorbance values were measured at varying time intervals at 620 nm. These samples were incubated so that the PRR agonist control reported an absorbance signal of approximately 1 A.U.

Viability Monitoring
Viability was monitored after overnight modulator addition by monitoring confluency via IncuCyte imaging. Two sets of parameters were used to create a confluency mask over all imaged wells. Modulators were considered toxic if both sets of confluency masks were < 70% of those of resting cells. Confluency masks were quantified using IncuCyte software. This methodology was validated with select library plates using a traditional CellTiter Glo assay (Promega). A more detailed description can be found in *SI Appendix,* Supplementary Information Text.

THP-1 Cell Culture
THP-1 cells were purchased from ATCC and were cultured in RPMI 1640 with 10% Biotin free HI-FBS and 1% P/S at 37 ˚C and 5% CO_2. Cells were maintained between 0.2-1.0 x 10^6 cells/mL and were not differentiated.

Cytokine Level Screening
THP-1 cells were seeded at 50,000 cells per well in 45 μL of biotin-free RPMI + 5%HI-FBS in clear 384 well plates. Following 24h incubation, 50 nL of 10 mM modulator libraries were added by Janus G3 via pintool to experimental wells (cols 3-22) for a final concentration of 10 μM. After 1 hour, 5 μL of agonist was added via a MultiDrop Combi liquid handler (col 3-23). The following day, 5 μL of supernatant was transferred to white low volume ProxiPlates (Perkin Elmer). According to AlphaPlex protocol, 10 μL of a prepared acceptor bead (10 μg/mL final conc), biotinylated antibody (1 nM final conc) mixture was added via liquid handler to the cell supernatant. After 1hr incubation at RT, 5 μL of donor beads (40 μg/mL final conc) were added in the dark. After an additional 1hr incubation, plates were read on a BioTek Synergy NEO2 plate

reader with AlphaPlex filters for europium (615 nm) and terbium (545 nm) emission. A separate plate was run each day containing a standard curve of known analyte for interpolation purposes.

BMDC Cytokine and Surface Marker Expression
Bone marrow was harvested from 6-week-old C57BL/6 mice and differentiated into dendritic cells (BMDCs) using supplemented culture medium: RPMI 1640 (Life Technologies), 10% HIFBS (Sigma-Aldrich), Recombinant Mouse GM-CSF (carrier-free)(20 ng/ml; BioLegend), 2 mM l-glutamine (Life Technologies), 1% antibiotic- antimycotic (Life Technologies), and 50μM β-mercaptoethanol (Sigma-Aldrich). After 6 days of culture, BMDCs were plated at 100,000 cells per well and incubated with modulator (10μM). After 1 hour, agonist was added. Cells were incubated for 24 hours at 37°C and 5% CO2. Supernatant cytokines were measured using LEGENDplex™ Mouse Inflammation Cytokine Kit (BioLegend). Cells were pelleted, washed, Fc blocked, and stained with the following antibodies/reagents at the recommended manufacturer's concentrations: CD11c-AF700 (N418), CD40-APC (3/23), I-A/I-E-AF488 (M5/114.15.2), CD86-BV605 (GL-1), CD80-PE (16-10A1), Live/Dead Fixable Blue. Data was acquired on a NovoCyte Penteon and analyzed via FlowJo 10.8.1.

NF-κB Dynamics
BMMΦ were generated from bone marrow isolated from 6-12 week old male RelA-YFP$^{+/+}$ C57BL/6 mice[50] and cultured in murine m-CSF (Peprotech) for 6-7 days. Custom 64 chamber automated microfluidic devices[51] were mounted on a Nikon Ti2 microscope and coated with fibronectin (Thermo Fisher). BMMΦ were loaded and allowed to adhere to the cell chambers for 5-7 hours. Cells were incubated with 1 μM Hoescht 33342 for 10 minutes to stain cellular nuclei for tracking. Hoescht and YFP images were taken every 6 minutes using excitation wavelengths of 395 nm and 508 nm with an exposure time of 50 ms or 1 s, respectively. During imaging, cells were automatically fed with either media or 10 μM modulator for two hours, followed by treatment with media, 10 μM modulator, or 10 μM modulator + R848. Cell videos were analyzed using custom MATLAB processing software[51] and single cells nuclear/cytoplasmic RelA ratios were tracked throughout the duration of the experiment. Single cell traces features were identified using the MATLAB function 'findpeaks', with a minimum prominence of 0.1 over the starting value and a minimum distance of 30 minutes between peaks. Microfluidic imaging protocol details can be found in Kellogg et al., 2014[42].

***In Vivo* Studies**
Animals
All animal procedures were performed under a protocol approved by the University of Chicago Institutional Animal Care and Use Committee (IACUC). 6-to-8-week-old C57/B6 female mice were purchased from the Jackson laboratory. All vaccinations were administered intramuscularly in the left hind leg. Blood was collected from the submandibular vein at time points indicated.

Vaccinations
VacciGrade Ovalbumin and Addavax was purchased from InvivoGen. VacciGrade CpG ODN 1826, ultrapure flagellin, and VacciGrade R848 were purchased from InvivoGen. Modulators were purchased through Selleck Chemicals.

Mice were lightly anesthetized with isoflurane and injected intramuscularly in the hind leg with an injection volume of 50 μL containing antigen, adjuvant, and a DMSO/Addavax combination. Antigen dose was as follows: OVA (100 μg). Agonist doses per mouse were as follows: Flagellin, 10 μg; R848, 50 μg; CpG, 50 μg. Modulators were added at 1.5 μmol. For commercial vaccines, 35μL of Typhim-Vi (Sanofi Pasteur) or Fluzone 2021-2022 (Sanofi Pasteur) were mixed with 15μL of DMSO with or without 1.5μmol of modulator for each mouse.

Plasma cytokine analysis
Blood was collected from mice at specified time points in 0.2 mL of heparin-coated collection tubes (VWR Scientific). Plasma was isolated via centrifugation 2000x*g* at 4C for 15 min. Samples were collected and stored at −80°C until use. Plasma was analyzed using Bio-Legend's

LEGENDPlex™ Mouse Inflammation Cytokine Panel (13-Plex) according to the manufacturer's protocol. Samples were analyzed using a NovoCyte Flow Cytometer. Data were analyzed using LEGENDplex™ Data Analysis Software Suite and GraphPad Prism.

Antibody quantification

Mice were vaccinated with indicated formulations. Blood was collected at time points indicated in 0.2 mL heparin-coated collection tubes (VWR Scientific). Plasma was isolated via centrifugation 2000x*g* at 4C for 15 min. Samples were collected and stored at −80 °C until use. Anti-ovalbumin antibody levels were analyzed using an anti-OVA IgG ELISA kit (Chondrex) according to the specified protocol. For influenza antibody levels, titers were performed by coating Nunc MaxiSorp ELISA plates (BioLegend) with 100µL of 2µg/mL hemagglutinin protein indicated (Sino Biological) overnight at 4C. Plates were washed and blocked with 150µL 2% (w/v) BSA in PBST for 1hr at RT. Following wash, samples were serially diluted tenfold in blocking buffer for a total volume of 100µL and incubated for 2hr at RT. Following wash, 100 µL of goat anti-mouse IgG HRP conjugate (Invitrogen) diluted 1:10,000 in 0.4% BSA buffer was incubated for 1hr at RT. Following wash, 100µL of 1-Step Ultra TMB was incubated for 4.5min before stopping with 50µL of 2M sulfuric acid. ELISA plates were analyzed using a Multiskan FC plate reader (Thermo Fisher) and absorbance was measured at 450 nm with 620nm correction. Area under the curve measurements were approximated with Riemann sums. Data were analyzed using GraphPad Prism.

Flow Cytometry for Germinal Center/Plasmablast Analysis

Mice were sacrificed 8 days after boost vaccinations with spleens harvested and placed on ice. Spleens were turned into single cell suspension by dissociation with two frosted glass slides and passage through a 70-µm filter. Red blood cells were lysed with 2mL ACK buffer (Gibco) for 3min before diluting, pelleting, washing, and resuspending in 1mL of RPMI media.

Statistics and replicates

Data are plotted and reported in the text as the means ± SD. Sample size is as indicated in biological replicates in all *in vivo* and *in vitro* experiments. The sample sizes were chosen based on preliminary experiments or literature precedent indicating that the number would be sufficient to detect significant differences in mean values should they exist. *P* values were calculated using a one-way analysis of variance (ANOVA) and Tukey post hoc test or two-tailed unpaired heteroscedastic *t* test where appropriate. Each approach is indicated alongside the experiment.

Key Resources Table

REAGENT or RESOURCE	SOURCE	IDENTIFIER
**Antibodies**		
Hamster Alexa Fluor® 700 anti-mouse CD11c Antibody	BioLegend	AB_528736 (BioLegend Cat. No. 117320)
Rat APC anti-mouse CD40 Antibody	BioLegend	AB_1134072 (BioLegend Cat. No. 124612)
Rat Alexa Fluor® 488 anti-mouse I-A/I-E Antibody	BioLegend	AB_493524 (BioLegend Cat. No. 107615)
Rat Brilliant Violet 605™ anti-mouse CD86 Antibody	BioLegend	AB_11204429 (BioLegend Cat. No. 105037)
Hamster PE anti-mouse CD80 Antibody	BioLegend	AB_313129 (BioLegend Cat. No. 104708)
Goat anti-Mouse IgG (H+L) Secondary Antibody, HRP	Thermo Fisher Scientfic	AB_228307 (ThermoFisher Cat. No. 31430)
Brilliant Violet 605™ anti-mouse CD138 (Syndecan-1) Antibody	BioLegend	AB_2715767 (BioLegend Cat. No. 142531)
APC/Cyanine7 anti-mouse CD4 Antibody	BioLegend	AB_312699 (BioLegend Cat. No. 100414)
TruStain FcX™ PLUS (anti-mouse CD16/32) Antibody	BioLegend	AB_2783138 (BioLegend Cat. No. 156604)
PE anti-mouse/human GL7 Antigen (T and B cell Activation Marker) Antibody	BioLegend	AB_2562926 (BioLegend Cat. No. 144608)
Alexa Fluor® 488 anti-mouse Ly-6A/E (Sca-1) Antibody	BioLegend	AB_493270 (BioLegend Cat. No. 108115)
PerCP anti-mouse CD19 Antibody	BioLegend	AB_2072926 (BioLegend Cat. No. 115532)
Brilliant Violet 785™ anti-mouse CD3 Antibody	BioLegend	AB_2562554 (BioLegend Cat. No. 100232
PE/Cyanine7 anti-mouse CD279 (PD-1) Antibody	BioLegend	AB_10696422 (BioLegend Cat. No. 135215)
BUV737 Hamster Anti-Mouse CD95	BD Biosciences	AB_2871122 (BD Biosciences Cat. No. 741763)_
BUV615 Rat Anti-Mouse CD185 (CXCR5)	BD Biosciences	Clone 2G8 (RUO) (BD Biosciences Cat. No. 752549)
**Bacterial and virus strains**		
Fluzone® Quadrivalent 2021-2022 Influenza Vaccine	Sanofi Pasteur	Cat# 635-15
**Chemicals, peptides, and recombinant proteins**		
LIVE/DEAD™ Fixable Blue Dead Cell Stain Kit	Invitrogen	Cat# L23105
Pam2CSK4	InvivoGen	Cat# tlrl-pm2s-1
Pam3CSK4	InvivoGen	Cat# tlrl-pms
Poly(I:C) HMW	InvivoGen	Cat# tlrl-pic
LPS-EK Ultrapure	InvivoGen	Cat# tlrl-peklps
MPLA-SM VacciGrade™	InvivoGen	Cat# vac-mpla
Flagellin FliC VacciGrade™	InvivoGen	Cat# vac-fla
Imiquimod (R837)	InvivoGen	Cat# tlrl-imq
R848 (Resiquimod)	InvivoGen	Cat# tlrl-r848-5
TL8-506	InvivoGen	Cat# tlrl-tl8506
Tri-DAP	InvivoGen	Cat# tlrl-tdap

MDP	InvivoGen	Cat# tlrl-mdp
3'3'-cGAMP	InvivoGen	Cat# tlrl-nacga-1
ODN 1826 (Type B)	AdipoGen	Cat# IAX-200-002-3001
Recombinant Mouse IFN-beta	Biotechne R&D Systems	Cat# 8234-MB-010/CF
NF-κB Signaling Compound Library	MedChemExpress	Cat# HY-L014
NF-κB Signaling Compound Library	Selleck Chemicals	Cat# L5500
Inhibitor Library	Selleck Chemicals	Cat# L1100
Typhim Vi® Polysaccharide Vaccine	Sanofi Pasteur	Cat# 790-20
Recombinant Hemagglutinin A/Victoria/2570/2019	Sino Biological	Cat# 40787-V08H
Recombinant Hemagglutinin B/Phuket/3073/2013	Sino Biological	Cat# 40498-V08B
EndoFit Ovalbumin	InvivoGen	Cat# vac-pova
AddaVax™	InvivoGen	Cat# vac-adx-10
Alexa Fluor™ 647 Microscale Protein Labeling Kit	Thermo Fisher Scientific	Cat#A300009
**Critical commercial assays**		
IFN-β (human) AlphaLISA Detection Kiti hav	Perkin Elmer	Cat# AL3133C
CCL4/MIP-1ß (human) AlphaLISA Detection Kit	Perkin Elmer	Cat# AL258C
IL-12/IL-23 (human) AlphaLISA Detection Kit	Perkin Elmer	Cat# AL382HV
AlphaPlex-545 (Tb) Human IP10 Detection Kit	Perkin Elmer	Cat# AP326TB-HV
AlphaPlex-545 (Tb) Human IL1β Detection Kit	Perkin Elmer	Cat# AP220TB-HV
AlphaPlex-545 (Tb) Human TNFα Detection Kit	Perkin Elmer	Cat# AP208TB-HV
LEGENDPlex™ Mouse Inflammation Cytokine Panel (13-Plex)	BioLegend	Cat# 740150
Mouse Anti-OVA IgG Antibody Assay Kit	Chondrex	Cat# 3011
**Experimental models: Cell lines**		
RAW-Dual Cells	InvivoGen	Cat# rawd-ismip
THP1-Dual Cells	InvivoGen	Cat# thpd-nfis
THP1 Cells	ATCC	Cat# TIB-202
**Experimental models: Organisms/strains**		
C57BL/6 mice	Jackson Laboratory	IMSR_JAX:000664
RelA-YFP$^{+/+}$ C57BL/6	Gift from Alex Hoffmann	
**Software and algorithms**		
NFKB dynamics processing software	Kellogg et al., 2014	N/A
GraphPad Prism	Dotmatics	https://www.graphpad.com/
FlowJo	Becton Dickinson	https://www.flowjo.com/

Supplementary Information Text

Cell Viability Analysis Via Confluency Masks

Cells were imaged using an IncuCyte S3 instrument (Sartorius). Confluency masks were created using the IncuCyte 2019B built-in analysis software. Two independent masks parameter sets were developed to better capture minute morphology differences between conditions.

Parameter set 1:
Segmentation Adjustment: 0.1
Cleanup
Hole Fill: 10μm^2, Adjust Size: 3 pixels
Filters
Area: min 1000 μm^2, Eccentricity: max 0.75

Parameter set 2:
Segmentation Adjustment: 1.0
Cleanup
Hole Fill: 0μm^2, Adjust Size: -5 pixels
Filters
Area: min 1000 μm^2, Eccentricity: N/A

Confluency percentages for all wells were calculated with both parameter sets and exported into Excel. Confluency for modulator treated wells was compared to the average confluency across all agonist-treated control wells. Modulators whose confluency measured less than 70% of the agonist-treated control wells were labeled cytotoxic. Modulators labeled cytotoxic in both parameter sets were removed from further study.

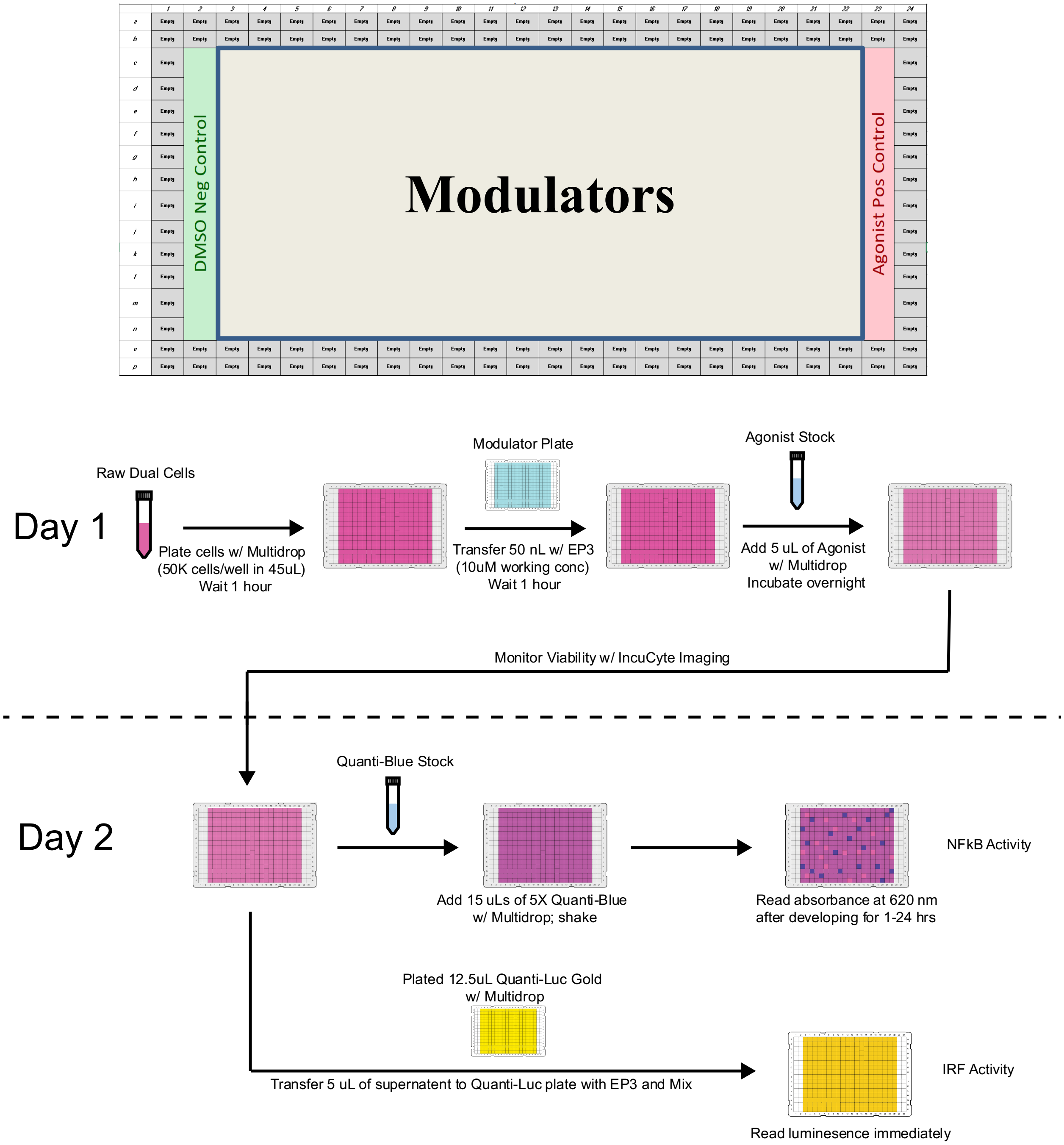

Fig. S1. High Throughput screening of NF-κB and IRF transcription factors. Layout of Primary Screen plates (N=14 plates) and schematic representation of the Primary Screen workflow.

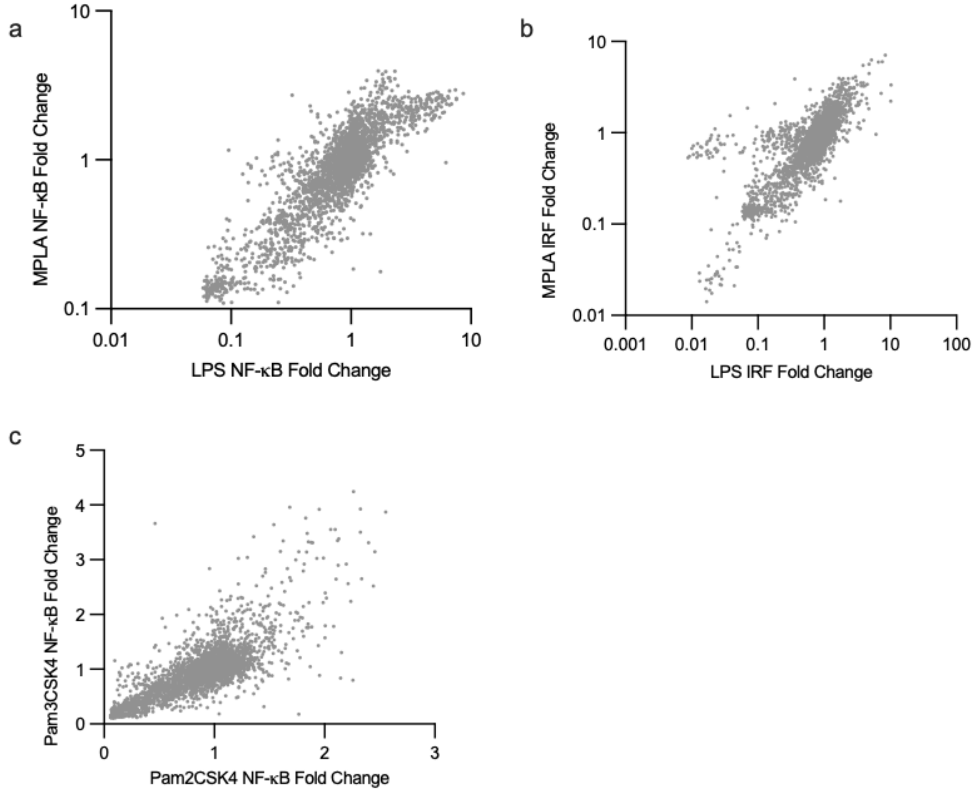

Fig. S2. Comparing NF-κB and IRF activity between similar agonists shows correlative activity. A) MPLA (TLR4) vs LPS (TLR4) NF-κB transcription factor fold change B) MPLA vs LPS IRF transcription factor fold change c) Pam2CSK4 (TLR2/6) versus Pam3CSK4 (TLR2/1) NF-κB transcription factor fold change

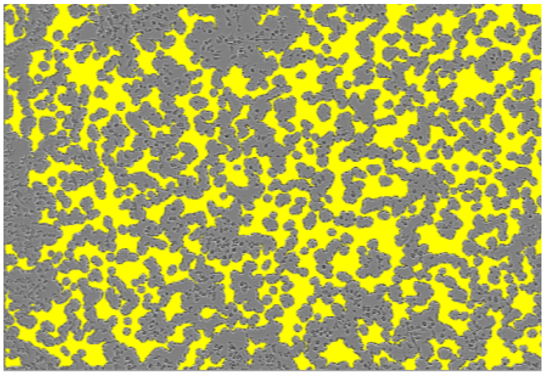

Parameter Set 1

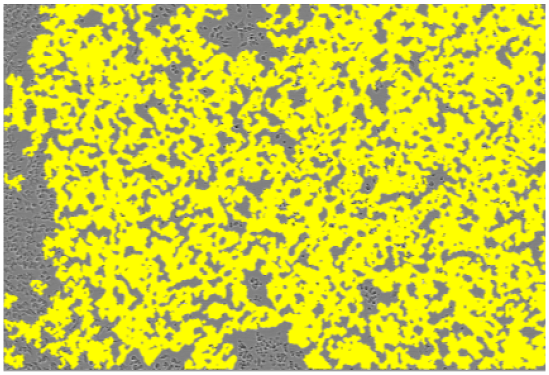

Parameter Set 2

	1	2	3	4	5	6	7	8	9	10	11	12	13	14	15	16	17	18	19	20	21	22	23	24
a	Empty	Empty	Empty	Empty	Empty	Empty	Empty	Empty	Empty	Empty	Empty	Empty	Empty	Empty	Empty	Empty	Empty	Empty	Empty	Empty	Empty	Empty	Empty	Empty
b	Empty	Empty	Empty	Empty	Empty	Empty	Empty	Empty	Empty	Empty	Empty	Empty	Empty	Empty	Empty	Empty	Empty	Empty	Empty	Empty	Empty	Empty	Empty	Empty
c	Empty	0	0	0	0	0	0	0	0	0	0	0	1	0	1	0	1	0	0	0	0	0	0	Empty
d	Empty	0	0	0	0	0	0	0	0	0	0	0	0	0	0	0	0	0	0	0	0	1	0	Empty
e	Empty	0	0	0	0	0	0	0	0	0	0	0	0	0	0	1	1	1	0	0	0	0	0	Empty
f	Empty	0	0	0	0	0	0	0	0	0	0	0	0	0	0	0	0	1	0	0	0	0	0	Empty
g	Empty	0	0	0	0	0	0	0	0	0	0	0	0	0	0	0	0	0	1	0	0	0	0	Empty
h	Empty	0	0	0	0	0	0	0	0	0	0	1	0	1	0	0	1	0	0	0	1	0	0	Empty
i	Empty	0	0	0	0	0	0	0	0	1	0	1	0	0	0	0	0	0	1	0	0	0	0	Empty
j	Empty	0	0	1	1	1	1	0	0	0	0	0	0	0	0	0	0	0	0	0	0	0	0	Empty
k	Empty	0	0	0	1	0	0	0	0	0	0	0	0	0	1	0	1	0	0	0	0	0	0	Empty
l	Empty	0	1	0	0	0	0	0	0	0	0	0	0	0	0	0	0	0	0	0	0	0	0	Empty
m	Empty	0	0	0	1	0	0	0	0	0	0	0	0	0	0	0	0	0	0	1	1	0	0	Empty
n	Empty	0	0	0	0	0	0	0	0	0	0	0	0	0	0	0	0	0	0	0	0	0	0	Empty
o	Empty	Empty	Empty	Empty	Empty	Empty	Empty	Empty	Empty	Empty	Empty	Empty	Empty	Empty	Empty	Empty	Empty	Empty	Empty	Empty	Empty	Empty	Empty	Empty
p	Empty	Empty	Empty	Empty	Empty	Empty	Empty	Empty	Empty	Empty	Empty	Empty	Empty	Empty	Empty	Empty	Empty	Empty	Empty	Empty	Empty	Empty	Empty	Empty

Fig. S3. Viability filter analysis identifies cytostatic and cytotoxic compounds in primary screen. A) Representative images of positive controls for both IncuCyte analysis parameter sets B) Representative library plate viability. Wells/compounds shown in red were determined as nonviable via the method described in the Supplemental Text.

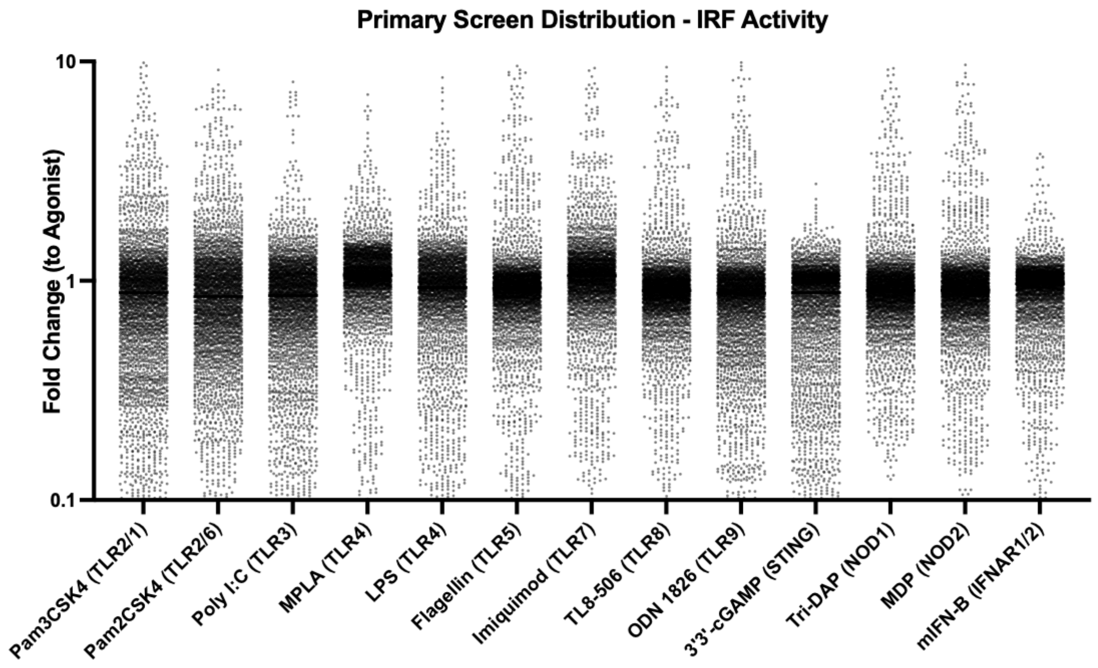

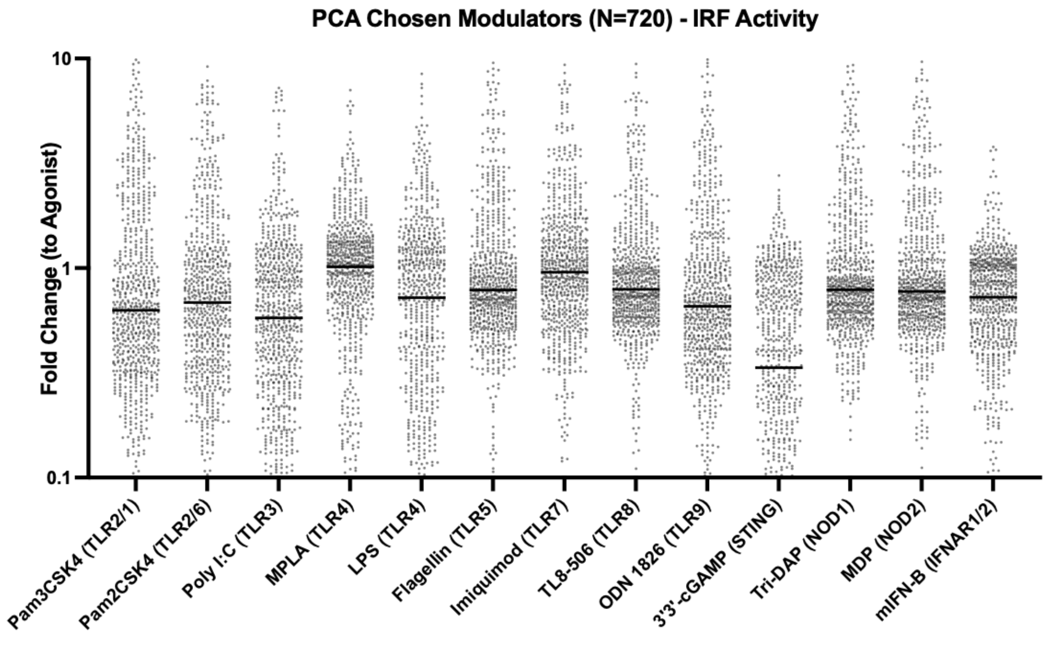

Fig. S4. Verification of PCA down-selection analysis. A) Full primary screen distribution (N=3147) for IRF transcription factor activity. B) Distribution of Modulators chosen by PCA cutting analysis (N=720) shows retention of tails of NF-κB and IRF distributions and a significant reduction of modulators around a fold change of 1.

a

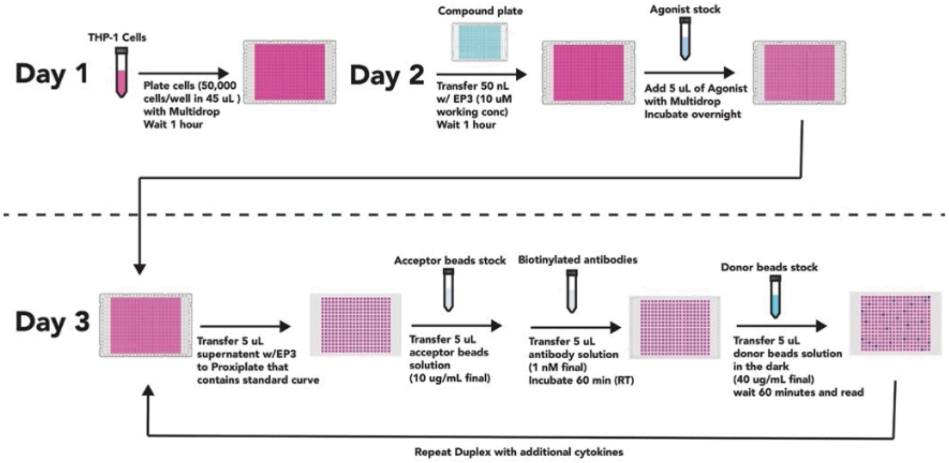

b

Tb Bead	Eu Bead
TNF-α	IFN-β
IL-1β	IL-12
IP-10	CCL4

c

	Tb >	Eu >
**Tb channel**	100.0%	0.42%
**Eu channel**	4.26%	100.0%

d

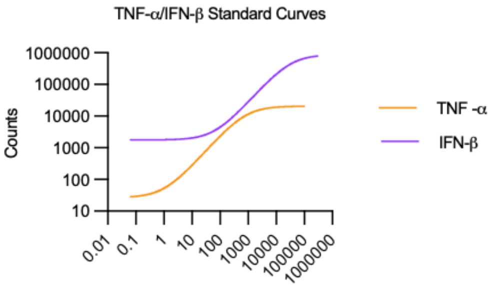

Fig. S5. Cytokine screen workflow and optimization. A) Schematic representation of the AlphaPlex workflow B) Table of beads for each cytokine duplex. C) Correction factors for terbium and europium emission needed for multiplexing cytokines. D) Representative standard curve used in interpolation

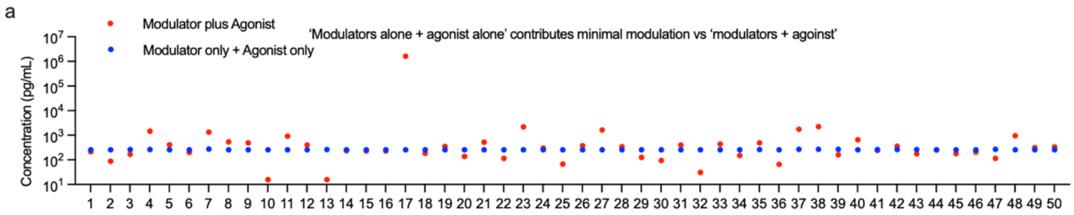

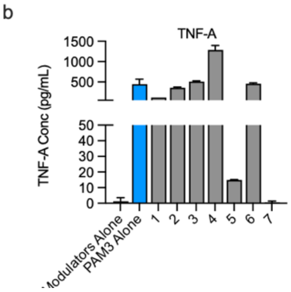

Fig. S6. Modulators alone do not inherently stimulate cytokine production. A) Representative data comparing the summation of modulator only + agonist only IL-1β expression levels (Blue) to modulator + agonist IL-1β expression (Red) with Pam3CSK4 (TLR2/1) as the agonist. B) Modulators alone produce minimal cytokine secretion (TNF-α shown). Addition of modulators to agonist modulate cytokine secretion over 3 orders of magnitude using Pam3CSK4.

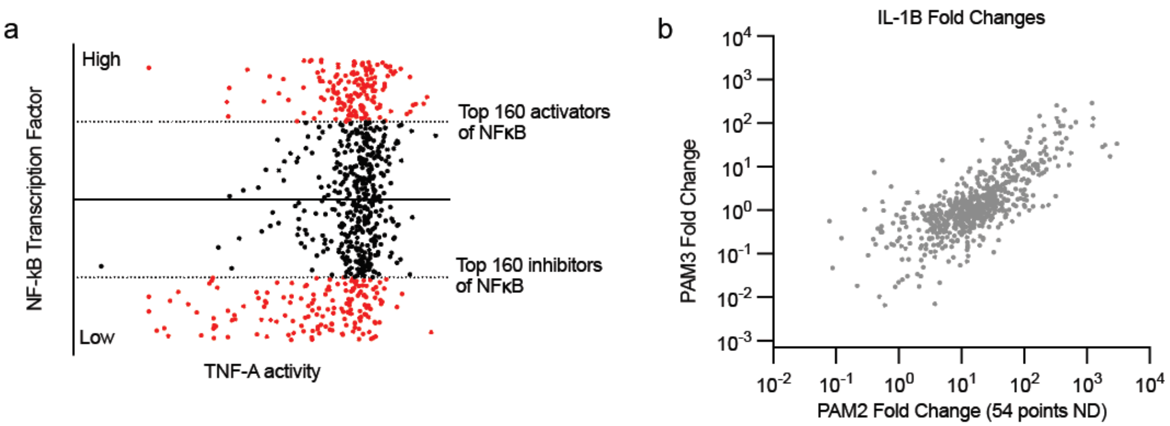

Fig. S7. Cytokine concentrations trends with transcription factor activity and agonist similarity A) For the top active compounds, transcription factor activity correlated with an increase of decrease cytokine response 10 B) IL-1β fold changes (N =720) of Pam2CSK4 versus Pam3CSK4 (Pearson R = 0.4507)
.

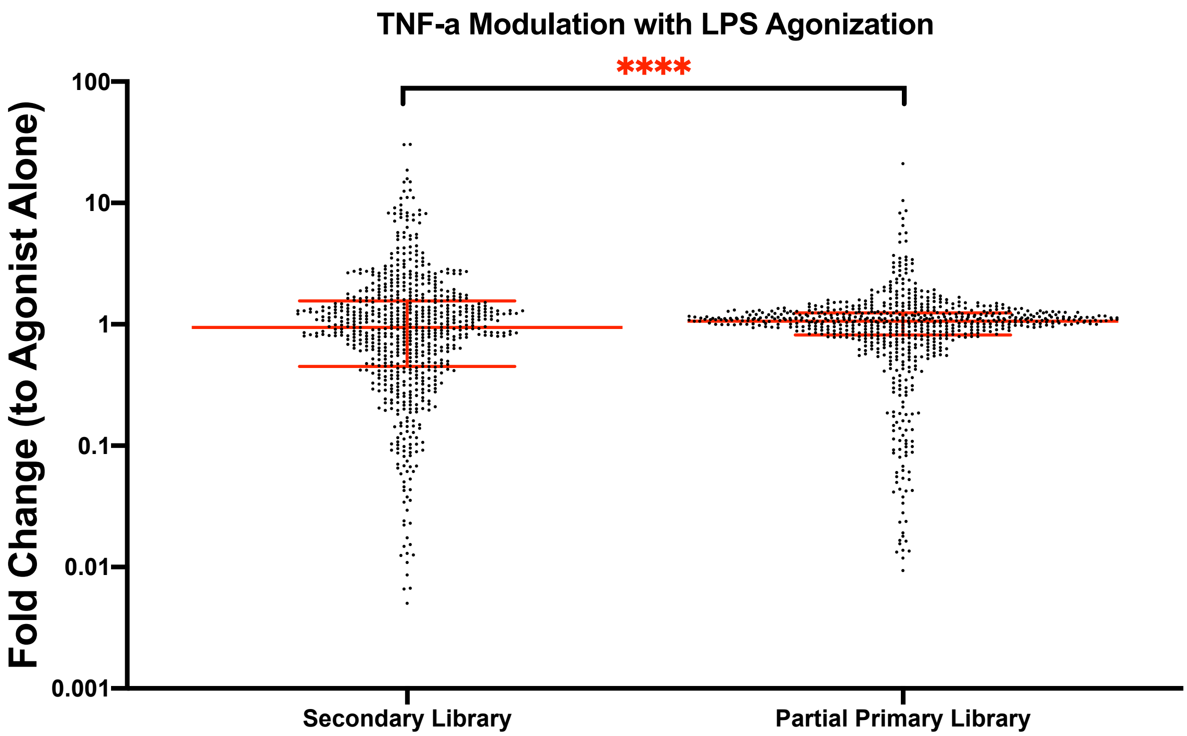

Fig. S8. Comparison of 720 PCA selected compounds to 720 random subsection of primary screen TNF-α comparison from a subsection of the primary screen (N=720) and the secondary library (N=720) show a significant increase in modulators that deviate from a fold change of one, showing evidence in the effectiveness of the PCA down selection method. ****P < .0001 Statistical analysis performed was a F Test to compare variance. Kurtosis of log-transformed primary screen: 6.884, secondary screen: 5.907.

	IL-1β	IL-12	IP-10	CCL4	TNF-α	IFN-β
Weight factor per cytokine (enhancer)	-0.5	1	1	0.5	-2	2
Weight factor per cytokine (inhibitor)	0.5	-1	-1	-0.5	2	0

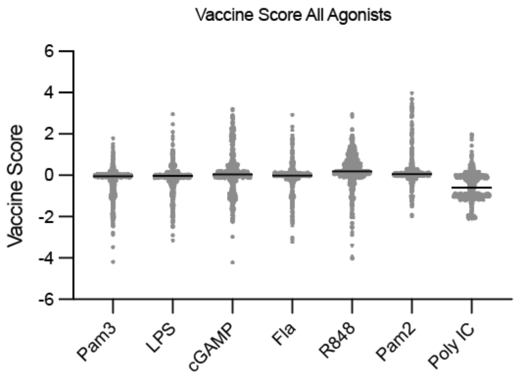

Fig. S9. Vaccine score generation. A) Table of weight factors for each cytokine for vaccine score B) Vaccine score for each agonist (N=720), individual agonist vaccine scores are summed up to create the generalist vaccine score.

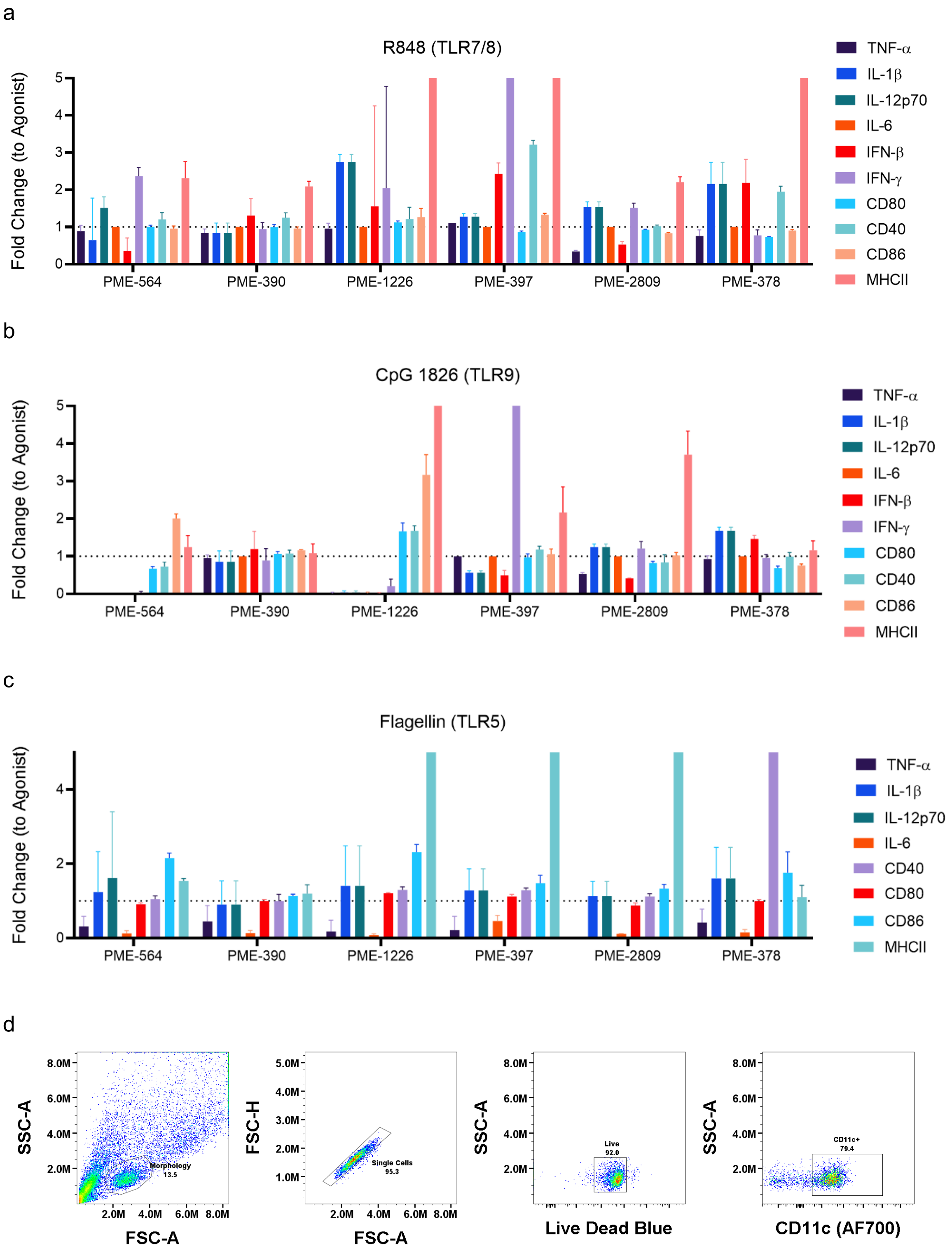

Fig. S10. BMDC immunomodulation distribution of top candidates from vaccine score
Cytokines and cell surface markers were measured 24 hours after agonizing with A) R848, B) Flagellin, and C) CpG. Modulators show significant ability to lower inflammatory cytokines in primary cells as well enhance cell surface markers. D) Gating strategy for flow cytometry

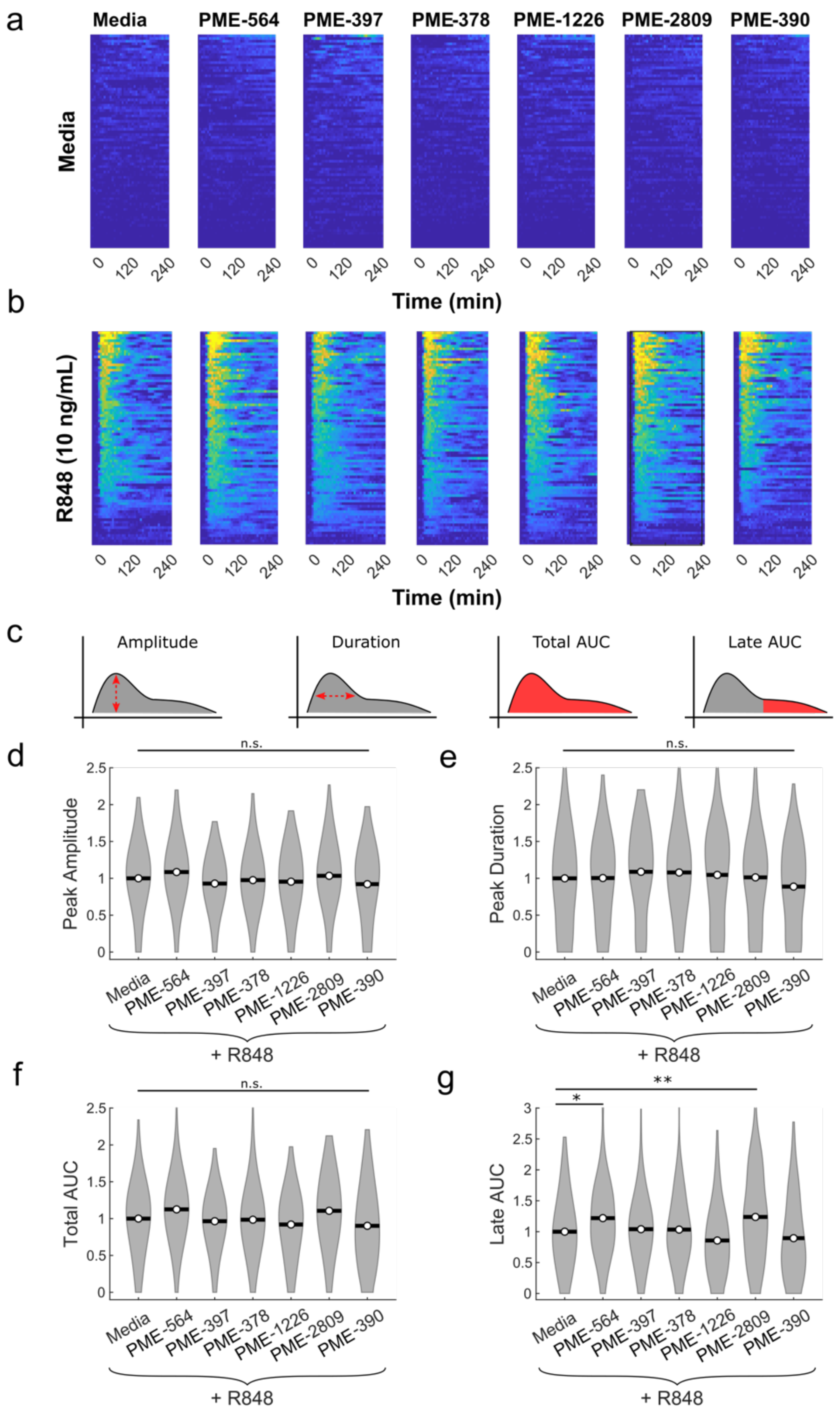

S11. Effect of top candidates on NF-κB activation dynamics. A-B) Heatmap of single cell NF-κB activation over time following treatment with modulators alone A) or modulators followed by 10 ng/mL R848 B). Each row is one of 80 randomly selected single cell traces for each condition. Data are from ≥ 90 single cells from two replicates. C) Schematic of NF-κB activation features measured for each trace. D-G) Violin plot showing distribution of single cell trace features in cells treated with top candidates following R848 stimulation. Black line and open circle show mean response. Measured features are D) amplitude, E) duration, F) total AUC, and G) late AUC as in c). * $p < 0.05$, ** $p < 0.01$, n.s. not significant using Bonferroni-corrected two-tailed t-test.

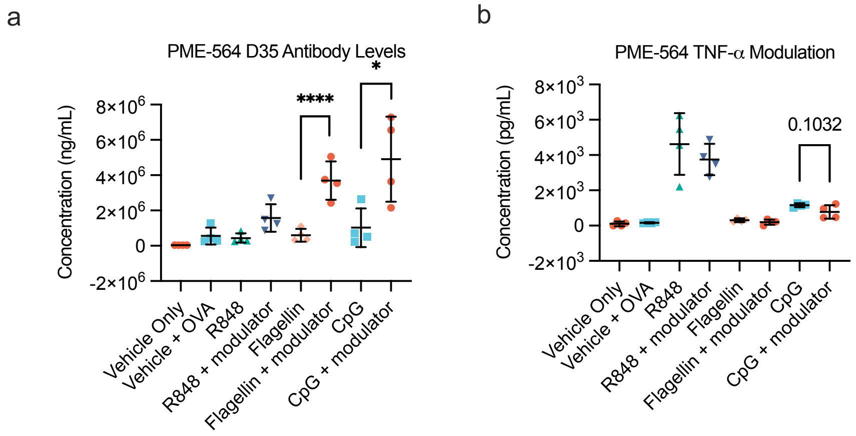

Fig. S12. ***In vivo*** **immunomodulation with PME-564 against OVA** A) PME-564 serum anti-OVA IgG antibody level, day 35, $n = 4$. B) Systemic TNF-α levels 1 hour after vaccination with agonist, agonist + modulator, and vehicle (N=4) for Flagellin (TLR5), R848 (TLR7/8) and CpG (TLR9). PME-564 statistical analyses between agonist and agonist + PME-564 were performed by an unpaired t test. *$P < 0.05$, **$P < 0.01$, ***$P < 0.001$, ****$P < 0.0001$. n.s., not significant.

a

Modulator	Name
1	PME-390
2	PME-1226
3	PME-397
4	PME-2809
5	PME-378

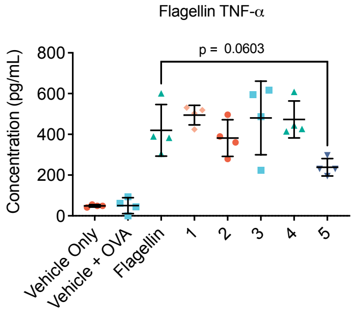

b

c

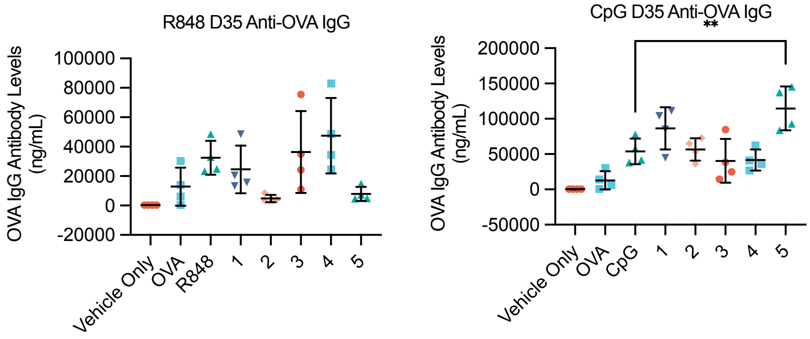

Fig. S13. *In vivo* immunomodulation of expanded generalist modulator panel. A) Compounds used for *in vivo* experiments. B) Systemic TNF-α levels 1 hour after vaccination with agonist, agonist + modulator, and vehicle (N=4) for Flagellin (TLR5) C) Modulator serum anti-OVA IgG antibody level for R848 (TLR7/8), and D) CPG (TLR9) on day 35, $n = 4$. Statistical analyses between agonist and agonist + modulator was performed by an unpaired t test. *$P < 0.05$, **$P < 0.01$, ***$P < 0.001$, ****$P < 0.0001$. n.s., not significant.

a

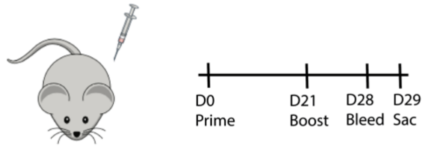
D0
Prime
D21
Boost
D28
Bleed
D29
Sac

b

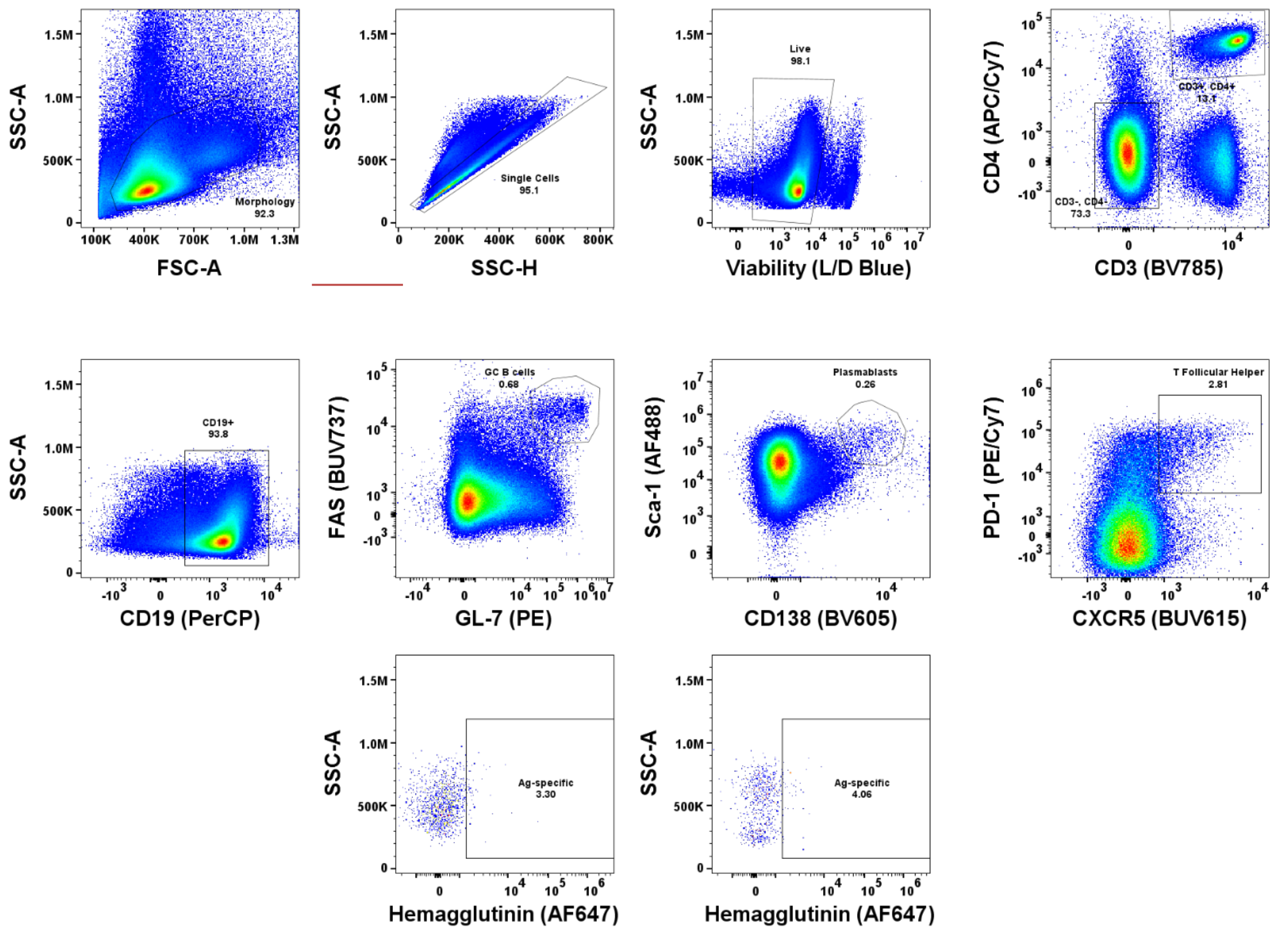
SSC-A
FSC-A
Morphology
92.3
SSC-H
Single Cells
95.1
Viability (L/D Blue)
Live
98.1
CD4 (APC/Cy7)
CD3 (BV785)
CD3+, CD4+
CD3-, CD4-
73.3
CD19 (PerCP)
CD19+
93.8
FAS (BUV737)
GL-7 (PE)
GC B cells
0.68
Sca-1 (AF488)
CD138 (BV605)
Plasmablasts
0.26
PD-1 (PE/Cy7)
CXCR5 (BUV615)
T Follicular Helper
2.81
Hemagglutinin (AF647)
Ag-specific
3.30
Hemagglutinin (AF647)
Ag-specific
4.06

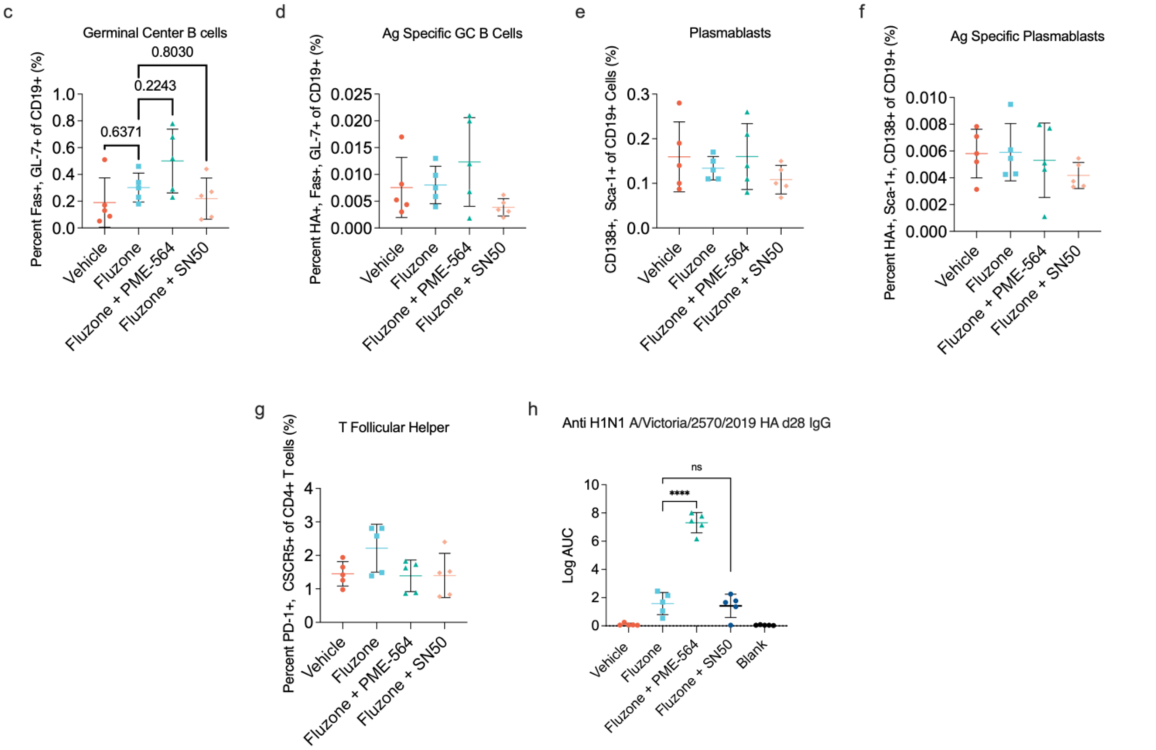

Fig. S14. Quantification of cell types critical for antibody production A) Schematic of *in vivo* study (N=5). B) Gating strategy for flow cytometry C/D/E/F/G) *In vivo* analysis of immune cells subsets. H) Modulator serum anti-OVA IgG antibody level on day 28. Statistical analyses between Fluzone + modulator groups and Fluzone alone were performed by a one-way ANOVA test *$P < 0.05$, **$P < 0.01$, ***$P < 0.001$, ****$P < 0.0001$. n.s., not significant.

a

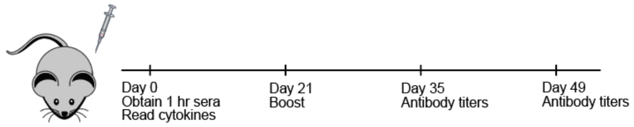

b

Modulator	Name
1	PME-390
2	PME-2583
3	PME-2988
4	PME-1653
5	PME-3035

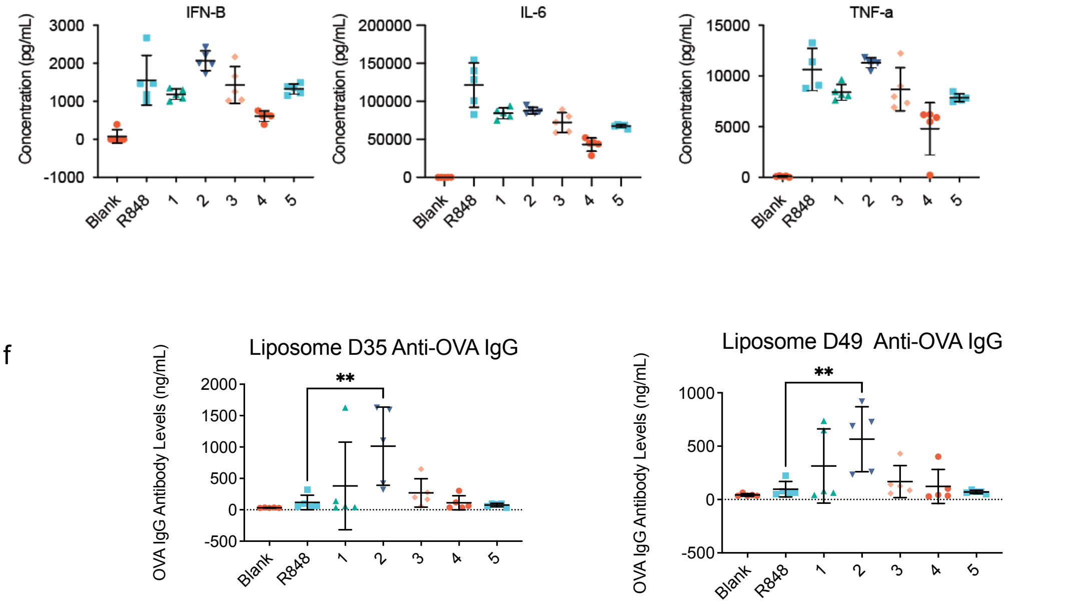

g

R848

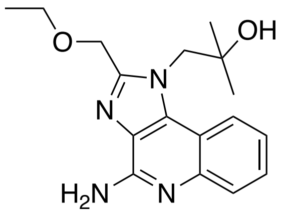

Fig. S15. Preliminary *in vivo* immunomodulation against OVA formulated in liposomes. A) Schematic of *in vivo* study B) Modulators studied *in vivo* were top performers in the constructed vaccine score C/D/E) *In vitro* IFN-β, IL-6, TNF-α levels 24 hour after addition with agonist in BMDCs. F) Modulator serum anti-OVA IgG antibody level on day 35 and 49. G) Structure of R848. Statistical analyses between agonist + modulator groups and agonist alone were performed by a one-way ANOVA test *$P < 0.05$, **$P < 0.01$, ***$P < 0.001$, ****$P < 0.0001$. n.s., not significant.

Table S1. Compounds from primary screen

Library	Source	Number
**Inhibitors**	Selleckchem	2790
**NF-κB Inhibitors**	Selleckchem	41
**NF-κB Inhibitors**	MedChem Express	206
**Tolerance**	Various	104
	**Total**	3141

Table S2. Agonists used in primary screen. Agonist targets, pathways activated, and working concentrations.

Agonist	*Target*	*NF-κB*	*IRF*	*Conc (ug/mL)*
Pam2CSK4	**TLR2/1**	**X**		**0.1**
Pam2CSK4	**TLR2/6**	**X**		**0.1**
Poly I:C	**TLR3**		**X**	**1**
MPLA	**TLR4**	**X**	**X**	**0.25**
LPS	**TLR4**	**X**	**X**	**0.1**
Flagellin	**TLR5**	**X**	**X**	**1**
Imiquimod	**TLR7**	**X**	**X**	**2.5**
TL8-506	**TLR8**		**X**	**0.1**
ODN 1826	**TLR9**	**X**	**X**	**0.5**
3'3'-cGAMP	**STING**		**X**	**10**
Tri-DAP	**NOD1**	**X**		**25**
MDP	**NOD2**	**X**		**10**
mIFN-β	**IFNAR1/2**	**X**	**X**	**0.00833**

Table S3. Z-factor analysis of primary screen agonists. High throughput screening utilizes Z-factors as a proxy for statistical reliability of an assay, due to the dynamic range of the positive controls and the inherent pathways for each agonist, a Z-factor score greater than zero was not obtained for certain agonists/transcription factor combinations**.**

Agonist	Target	NF-κB Activator	NF-κB Z- factor	IRF Activator	IRF Z- factor
**Pam3CSK4**	TLR 2/1	x	0.193448		<0
**Pam2CSK4**	TLR 2/6	x	0.509904		<0
**Poly I:C**	TLR3		<0	x	<0
**MPLA**	TLR4	x	0.729615	x	0.668819
**LPS**	TLR4	x	0.330641	x	0.425865
**Flagellin**	TLR5		<0		<0
**Imiquimod**	TLR7	x	0.826412		<0
**TL8-506**	TLR8		<0		<0
**ODN 1826**	TLR9	x	0.785698	x	<0
**3'3'-cGAMP**	STING		<0	x	0.699694
**Tri-DAP**	NOD1		<0		<0
**MDP**	NOD2		<0		<0
**mIFN-β**	IFNAR1/2		<0	x	<0

Table S4. **Cytokines measured in secondary screen.** Functions of cytokines chosen for secondary screen in AlphaPlex investigation

Cytokine	*Function*
TNF-α	Promotes inflammation
IL-1β	Fever, partially outside NF-κB
IFN-β	Antiviral, increased MHCI
IL-12/23 (p40)	Activates NK cells, induces IFN-γ, roles in T-cell polarization
IP-10 (CXCL10)	Chemoattractant for T cells, DCs
CCL4	Chemoattractant for monocytes, NK cells, others

Table S5. Z-factor analysis of secondary screen. High throughput screening utilizes Z-factors as a proxy for statistical reliability of an assay, due to the dynamic range of the positive controls and the inherent cytokines secreted for each agonist, a Z-factor score greater than zero was not obtained for certain agonists/transcription factor combinations.

			**Cytokine**					
**Agonist**	**Target**	**Conc (μg/mL)**	IL-1β	IL-12	IP-10	CCL4	TNF-α	IFN-β
**Pam3CSK4**	TLR2/1	0.1	.51	.67	.23	.82	.68	-
**Pam2CSK4**	TLR2/6	0.1	-.76	.69	.12	.62	.66	-
**LPS**	TLR4	0.1	.69	.32	.50	.67	.71	-
**Flagellin**	TLR5	1	.03	.60	-.19	.57	.71	-
**R848**	TLR7	10	.79	.88	.87	.56	.61	-.6
**3'3'-cGAMP**	STING	10	-	-	-	-	-.69	-
**Poly IC**	TLR3	1	-	-	-	-	-	-
**Mods alone**								
