# Supplementary material for: Discovery of New States of Immunomodulation for Vaccine Adjuvants via High Throughput Screening: Expanding Innate Responses to PRRs
Source: ACS Cent Sci. 2023 Feb 23;9(3):427–39. doi: 10.1021/acscentsci.2c01351 (PMC10037445; doi:10.1021/acscentsci.2c01351)
Supplement: Supplementary file 3 — oc2c01351_si_003.pdf [file oc2c01351_si_003.pdf]

Overall

Thank you for the formatting changes, all suggested changes have been fixed. A supporting information section has been included in the main text, and is copied below:

Supporting Information

The supporting information includes detailed methods, key resources tables, supplementary text, figures S1 to S15, and Table S1 to S5. SI figures include high throughput workflow and optimization, BMDC immunomodulation experiments, NF-κB activation dynamics, flow cytometry analysis, extended datasets, and preliminary liposome experiment data. SI tables include compound library sources, agonist concentrations, and high throughput optimization.

Reviewer 1

1. The authors should perform a direct comparison of their new method to prior described approaches that utilize small molecules to modulate vaccine responses using the key readouts presented in the main text. These new data should be included in the main figures to demonstrate how effective their newly reported approach is compared to existing technologies.

   In Figure 5, we show the effects of adding our modulators compared to the effects of R848, a small molecule, and CpG 1826, a short oligodeoxynucleotide, in a subunit vaccine. This is a direct comparison between prior immunostimulatory small molecules and our approach which uses the combination of an immunostimulatory molecule, and a separate modulator. Alone, our modulators are not immunostimulatory, and thus our approach is unique and difficult to directly compare responses. To our knowledge, the only prior approach of modulating adjuvant signaling with small molecules is our published work. We cite our prior studies in this work, and thus did not think it necessary to include prior data in the main figures.

2. It would also be worth discussing the literature precedence of using small molecules to influence vaccine responses in the introduction of the manuscript (and cite these references) and to comment on how their approach compares to these existing methods (pros and cons) in the introduction and discussion sections.

   Added a sentence in the introduction describing the difference between prior high throughput screens for small molecule agonists and our new small molecule immunomodulators. Provided three citations.

   We moved a paragraph describing PME-564 from results to the discussion and added description and details of other top immunomodulators used. In the discussion, we also broadly recap how our use of small molecules to modulate signaling after agonist recognition compares to the use of small molecules as agonists alone. We also compared early antibody levels (d28) between PME-564 and SN50, a previously studied immunomodulator, and saw that addition of PME-564 yielded higher antibodies (*SI Appendix*, Figure S14).

3. The authors should perform FACS analysis of certain immune cells that are essential to antibody production (e.g., B cells and CD8 T cells) in their in vivo models presented in Fig 6, which is conventional for vaccine studies. For example, can they quantify plasma B cells that secrete antibodies in the appropriate tissues to demonstrate that B cell responses are responsible for the increased antibody levels?

   We performed flow cytometry analysis focusing on plasmablasts, germinal center B cells, and T follicular helper cells, but did not find any significant difference between Fluzone alone and Fluzone with modulator. Thus, we added this data to the *SI Appendix*, Figure S14. The gating strategy can be seen, as well as the percentages for each of the studied phenotypes. A few sentences have been added to the vaccination portion of the Results section.

Reviewer 2

1. The authors purchased the 3,000 small molecules from vendors. No mention of how the compound libraries were assessed for purity/decomposition – both of which are important. The authors did test that the modulators do not provide activity on their own (Figure 2). However, nothing is done to assure that the compounds are pure and the true source of alerting the activity of the agonist.

   It is true we did not assess the small molecule libraries for purity or decomposition. These two issues are a concern in high throughput screening. The vast majority of our compounds were sourced through Selleck, and an independent *Nature* study found Selleck had the highest percentage of pure compounds from a variety of tested vendors. We used the compound libraires within a year of purchase, the vendors claim stability at -80C for two years. The vendor states libraries are quality controlled for purity through HPLC and NMR, but statistics for each compound in the library are not available. However, for our low throughput *in vitro* and all *in vivo* experiments, we purchased additional stocks of small molecules, separate from the libraries. These individual stocks do have lot-specific NMR spectra and HPLC traces, confirming purity of >99%. We believe effects from the high throughput screening stages to be confirmed through *in vivo* experimentation.

2. As this is a chemical journal, structures of the compounds – especially those used in the animal- vaccination studies should be shown.

   We have added chemical structures for all compounds used in the animal experiments in the main text. Structures for all non-toxic compounds that were not used in the main text can be found as SMILES in the attached library datasheet.

3. The method section is well written. For THP cells (supporting information), where the cells differentiated prior to use? If so, please describe conditions.

The THP-1 cells were not differentiated prior to use; this has been made clear in the methods section.

4. For the improving the vaccination responses in mice, the authors chose a modulator dosage of 1.5 uM. How does this range compare to the cell studies? And if an increase, could the authors comment if it is necessary to repeat the cell based assays?

   We chose an immunomodulator dosage of 1.5μmol per mouse in the vaccine studies. With a 50μL injection, the concentration in the syringe is much higher than the cell based studies. We hypothesized that poor delivery and solubility, however, would result in a local concentration seen by an immune cell once injected into the tissue. We have since performed dose response studies in vivo and found we must give high doses to see an effect. We chose our initial dose based off previously published literature for small molecule immunomodulators. We do not believe it necessary to repeat the cell based assays – the screening concentration of 10μM is likely higher than the local dose given *in vivo.*

5. The authors briefly mention that solubility of the modulatory is important and liposomal delivery systems diminished activity. I think it would be very helpful if the structures of the modulators and agonist were shown in the SI.

   Structures of compounds used in any animal experiment including liposome experiment can be found in the main text or in the library information dataset (as SMILES). R848's structure was added to S15.

Thank you for your thoughts,

Aaron Esser-Kahn
